# Longitudinal dynamics of clonal hematopoiesis identifies gene-specific fitness effects

**DOI:** 10.1038/s41591-022-01883-3

**Published:** 2022-07-04

**Authors:** Neil A. Robertson, Eric Latorre-Crespo, Maria Terradas-Terradas, Jorge Lemos-Portela, Alison C. Purcell, Benjamin J. Livesey, Robert F. Hillary, Lee Murphy, Angie Fawkes, Louise MacGillivray, Mhairi Copland, Riccardo E. Marioni, Joseph A. Marsh, Sarah E. Harris, Simon R. Cox, Ian J. Deary, Linus J. Schumacher, Kristina Kirschner, Tamir Chandra

**Affiliations:** 1 MRC Human Genetics Unit, University of Edinburgh, Edinburgh, UK; 2 Institute of Cancer Sciences, College of Medical, Veterinary and Life Sciences, University of Glasgow, Glasgow, UK; 3 Cancer Research UK Beatson Institute, Glasgow, UK; 4 Centre for Regenerative Medicine, Institute for Regeneration and Repair, University of Edinburgh, Edinburgh, UK; 5 Centre for Genomic and Experimental Medicine, Institute of Genetics and Cancer, University of Edinburgh, Edinburgh, UK; 6 Edinburgh Clinical Research Facility, University of Edinburgh, Edinburgh, UK; 7 Paul O’Gorman Leukaemia Research Centre, Institute of Cancer Sciences, College of Medical, Veterinary and Life Sciences, University of Glasgow, Glasgow, UK; 8 Lothian Birth Cohorts, Department of Psychology, University of Edinburgh, Edinburgh, UK

**Keywords:** Cancer genomics, Haematopoietic stem cells, Risk factors, Haematological cancer, Cancer stem cells

## Abstract

Clonal hematopoiesis of indeterminate potential (CHIP) increases rapidly in prevalence beyond age 60 and has been associated with increased risk for malignancy, heart disease and ischemic stroke. CHIP is driven by somatic mutations in hematopoietic stem and progenitor cells (HSPCs). Because mutations in HSPCs often drive leukemia, we hypothesized that HSPC fitness substantially contributes to transformation from CHIP to leukemia. HSPC fitness is defined as the proliferative advantage over cells carrying no or only neutral mutations. If mutations in different genes lead to distinct fitness advantages, this could enable patient stratification. We quantified the fitness effects of mutations over 12 years in older age using longitudinal sequencing and developed a filtering method that considers individual mutational context alongside mutation co-occurrence to quantify the growth potential of variants within individuals. We found that gene-specific fitness differences can outweigh inter-individual variation and, therefore, could form the basis for personalized clinical management.

## Abstract (web summary)

An analysis of blood samples from longitudinal cohorts reveals insights on the dynamics of clonal hematopoiesis of indeterminate potential and proposes a model that could be used for individualized clone monitoring over time.

## Main

Age is the single largest factor underlying the onset of many cancers^1^. Age-related accumulation and clonal expansion of cancer-associated somatic mutations in healthy tissues has been posited recently as a pre-malignant status consistent with the multi-stage model of carcinogenesis^2^. However, the widespread presence of cancer-associated mutations in healthy tissues highlights the complexity of early detection and diagnosis of cancer^3–7^.

CHIP is defined as the clonal expansion of HSPCs in healthy aged individuals. CHIP affects more than 10% of individuals over the age of 60 years and is associated with an estimated ten-fold increased risk for the later onset of hematological neoplasms^3–5^. There is a clear benefit of detecting CHIP early for close clinical monitoring and early detection, as the association between clone size and malignancy progression is well-established^5,8,9^.

The particular mechanisms by which common mutations of CHIP—for example, *DNMT3A* and *TET2*—contribute to the progression of leukemia are still not understood, which hinders early diagnosis of CHIP on a gene or variant basis^8,10–12^. In clinical practice, CHIP is diagnosed by the presence of somatic mutations at variant allele frequencies (VAFs) of at least 2% in cancer-associated genes, that is in more than 4% of all blood cells^8,13^. Clonal fitness, defined as the proliferative advantage of stem cells carrying a mutation over cells carrying no or only neutral mutations, has emerged as an alternative clone-specific quantitative marker of CHIP^14,15^. As mutations in stem cells often drive leukemia^5^, we hypothesized that stem cell fitness contributes substantially to transformation from CHIP to leukemia.

Stratification of individuals to inform close clinical monitoring for early detection or prevention of leukemia in the future will depend on the ability to accurately associate genes and their variants with progression to disease. However, it remains unresolved whether variant-specific or gene-specific fitness effects outweigh other factors contributing to variable progression among individuals, such as environment or genetics.

Hitherto, fitness effects have been predicted from large cross-sectional cohort data^14,16^. In this approach, single-timepoint data from many individuals are pooled to generate allele frequency distributions. Although this method allows the study of a large collection of variants, pooling prevents estimation of an individual’s mutational fitness effects from cross-sectional data. Inferring fitness from a single timepoint creates additional uncertainty about whether a mutation has arisen recently and has grown rapidly (high fitness advantage) or arose a long time ago and has grown slowly (low fitness advantage). With longitudinal samples, fitness effects of individual mutations can be estimated directly from the change in VAF over multiple timepoints.

In this study, we worked with longitudinal data from the Lothian Birth Cohort of 1921 (LBC1921) and the Lothian Birth Cohort of 1936 (LBC1936)^17^. Such longitudinal data are rare worldwide owing to their participants’ older age (70–90 years) and their three-yearly follow-ups over 12 years in each cohort and over 21 years of total timespan. We developed a new framework for extracting fitness effects from longitudinal data using Bayesian inference. First, a likelihood-based filter for time series data (LiFT) allowed us to segregate between sequencing artifacts or naturally drifting populations of cells and fast-growing clones. Second, we inferred the growth potential or fitness effects simultaneously for all growing mutations within each individual and also allowed for clones with multiple mutations if these are favored by Bayesian model comparison. We detected gene-specific fitness effects within our cohorts, highlighting the potential for personalized clinical management.

## Results

### Longitudinal profiling of CHIP variants in advanced age

**Extended Data Fig. 1:**
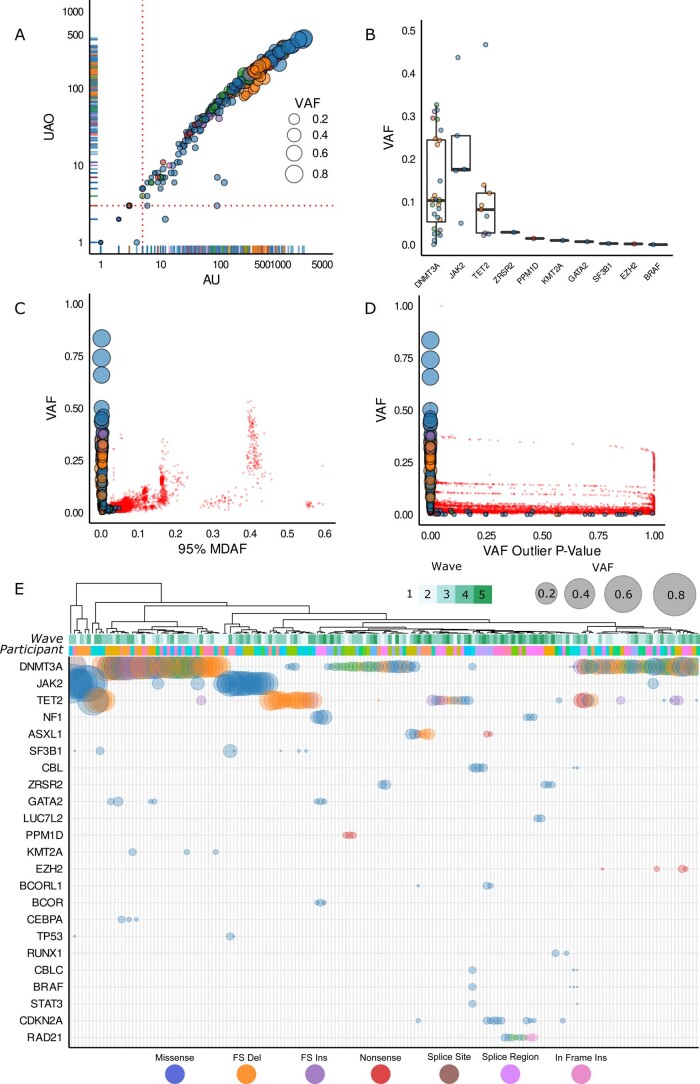
Quality Control Metrics. **a**. Sequence quality metrics for mutation calls across participants and time-points filtered for 2% VAF. Plotted are the AO (the number of sequenced reads supporting the alternative allele (mutation)) against the UAO (the number of sequenced reads with unique start sites that support the alternative allele - a measure of molecular complexity). Red dotted lines denote filter thresholds in both measurements (AO ≥ 5, UAO ≥ 3) and points are scaled by the VAF of the somatic mutation. Only 7 (of 275) data points failed to meet our filter criteria which were not excluded as they were supported with matching events across any participants’ time series. **b**. Box and jitter plot of the variant allele frequency of all observed events in the 1st Wave at 2% VAF coloured by variant classification and ordered by largest mean VAF showing the median and interquartile range. **c**. The 95% MDAF (Minimal Detectable Allele Fraction with 95% Confidence) versus the VAF for each event. All variants used in our analysis above 2% VAF are scaled by their clone size and coloured by their functional consequence. Points in red are events that failed to pass our quality criteria and are removed from subsequent work. **d**. The VAF Outlier P-Value (describing the pan-cohort position-specific background noise) versus VAF for each event. All variants used in the analysis above 2% VAF are scaled by their clone size and coloured by their functional consequence. Points in red are events that failed to pass our quality criteria and are removed from subsequent work. All accepted events that exceed VAF Outlier P-Value > 0.1 are generally low VAF and are supported by matching events across the time-series that adhere to our acceptance criteria of VAF Outlier P-Value ≤ 0.1. **e**. Schematic of all affected genes in the cohort with the largest clone size of an event in any given gene shown above 2% VAF. All affected participants have been clustered across all time-points, with the point size scaled by VAF and coloured by the functional consequence of the variant (as per legend).

The Lothian Birth Cohorts (LBCs) of 1921 (*n* = 550) and 1936 (*n* = 1091) are two independent, longitudinal studies of aging with approximately three-yearly follow-up for five waves, from the age of 70 years (LBC1936) and 79 years (LBC1921)^17^. We previously identified 73 participants with CHIP at wave 1 through whole-genome sequencing (WGS)^18^. Here, we used a targeted error-corrected sequencing approach using a 75-gene panel (ArcherDX/Invitae) to assess longitudinal changes in VAFs and clonal evolution over 21 years across both LBC cohorts (6 years in LBC1921 and 12 years in LBC1936; Supplementary Table 1). Error-corrected sequencing allowed accurate quantification, providing more sensitive clonal outgrowth estimates than our previous WGS data. We sequenced 248 LBC samples (85 individuals across 2–5 timepoints) and achieved a sequencing depth of 2,238× mean coverage (2,153× median) over all targeted sites with an average of 1.6 unique somatic variants (pan-cohort VAF 0.03–87%, median VAF 4.4%) detected per participant. We examined all participant-matched events across the time course: sequence quality control metrics revealed that only seven of 275 data points failed to meet our quality criteria, likely due to low initial VAF. Most of our variant loci generally displayed a high number of supporting reads, with a mean of 258 (Extended Data Fig. 1a).

**Fig. 1:**
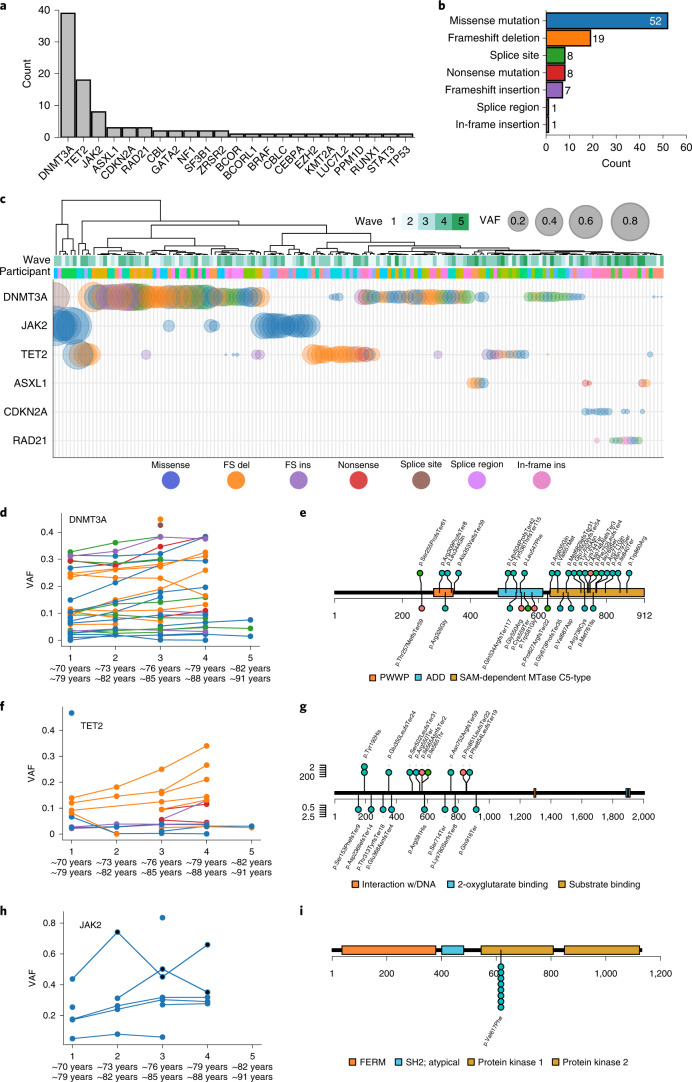
Clonal hematopoiesis in the LBCs. **a**, Counts of unique events that exceeded 2% VAF across the range of the longitudinal cohorts in our panel of 75 hematopoietic genes. **b**, Counts of the functional consequences of the unique events listed in Fig. 1a. Missense mutations, frameshift insertions and deletions and nonsense mutations are indicated. Exact counts, *n*, are for each category. **c**, Schematic of the top seven most affected genes in the cohort with the largest clone size of an event in any given gene shown. All affected participants were clustered across all timepoints, with the point size scaled by VAF and colored by the functional consequence of the variant (as per Fig. 1b and legend). **d**, Clone size trajectories of all *DNMT3A* mutations across the time series in both LBC1921 and LBC1936 colored by the functional consequence of the variant (as per Fig. 1b,c). **e**, Locations of somatic mutations discovered in *DNMT3A*. Protein-affecting events are marked and labeled across the structure of the gene (missense in red, truncating in purple, stacked for multiple events) with the structure of the gene labeled along the amino acid length of its protein. **f**. Clone size trajectories of all *TET2* mutations across the time series in both LBC1921 and LBC1936 colored by the functional consequence of the variant (as per Fig. 1b,c). **g**, The locations of somatic mutations in *TET2*. Protein-affecting events are marked and labeled across the structure of the protein (missense in red, truncating in purple, stacked for multiple events). **h**, Clone size trajectories of all *JAK2* mutations across the time series in both LBC1921 and LBC1936 colored by the functional consequence of the variant (as per Fig. 1b,c). Points marked in black denote timepoints after which the affected participant received treatment for leukemia. **i**, The locations of somatic mutations in *JAK2*. Protein-affecting events are marked and labeled across the structure of the protein (missense in red, truncating in purple, stacked for multiple events). All eight *JAK2* mutations are p.Val617Phe (*JAK2 V617F*) missense variants. del, deltion; FS, frameshift; ins, insertion.

For our initial analysis, we retained variants with at least one timepoint at 2% VAF (Supplementary Table 2). *DNMT3A* was the most commonly mutated CHIP gene (*n* = 39 events in 33 participants), followed by *TET2* (*n* = 18 events in 15 participants), *JAK2* (*n* = 8 events in eight participants) and *ASXL1* (*n* = 3 events in three participants) (Fig. 1a–c and Extended Data Fig. 1e). Our mutation spectrum is consistent with previous studies in finding *DNMT3A* and *TET2* as the most frequently mutated genes^4,5^. We detected some variants more frequently at certain hotspots within a gene, such as R882H in *DNMT3A*, with previously unreported variants being present as well (Fig. 1d–i and Supplementary Table 2)^5^. We most frequently detected missense mutations with several other key protein-altering event types ranking highly, including frameshift insertions and deletions and nonsense mutations (Fig. 1a–c). Participants broadly cluster together across their time course, driven by the expanding or stable VAF of their harbored mutations, underscoring the high prevalence and large clone size of common clonal hematopoietic drivers, namely *DNMT3A*, *TET2* and *JAK2* (Fig. 1a–c). In the case of *JAK2V617F*, we identified two individuals who developed leukemia at wave 2 and received treatment between waves 2 and 3, likely driving a clear reduction in clone size (Fig. 1h). Those individuals were excluded from further analysis. In our data, we identified a lower frequency of mutations in splicing genes, such as *SF3B1*, despite the older age of the cohorts (Fig. 1a and Extended Data Fig. 1e). This is in contrast to previously published cohort data, where splicing mutations became more prominent with increased age^19^. Most mutations were missense, frameshift and nonsense mutations (Fig. 1b).

Overall, our sequencing approach allowed for high-resolution, longitudinal mapping of CHIP variants over 6-year and 12-year time spans in LBC1921 and LBC1936, respectively, and 21-year time span across both cohorts from the same geographical region and born 9 years apart.

### Cataloguing of fitness effects for CHIP variants at >2% VAF

**Fig. 2:**
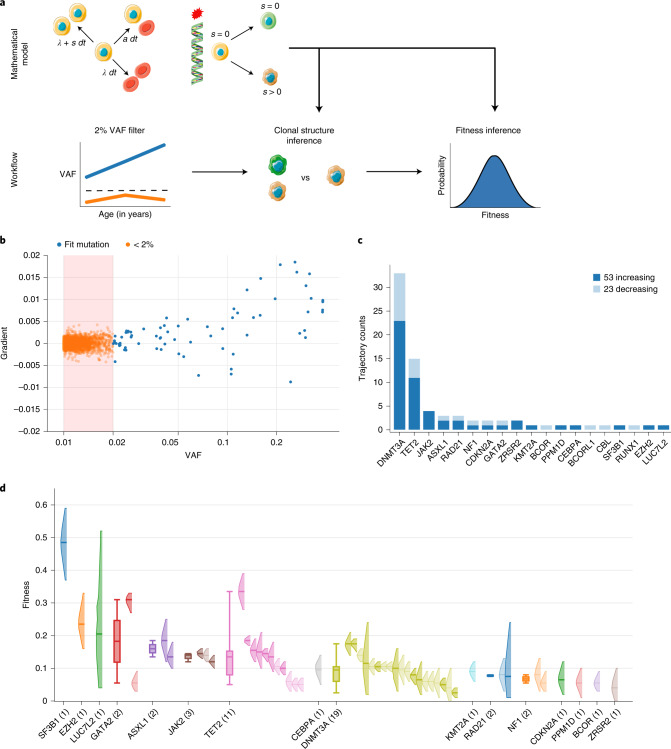
Fitness effects of variants at 2% VAF threshold in longitudinal data. **a**, Schematic of the mathematical model (top) and workflow (bottom) used to infer the fitness of mutations reaching VAF > 2% during the observed time span. Clonal structure and fitness inference are based on a mathematical model of clonal dynamics (Methods). HSPCs (top, yellow cells) naturally acquire mutations over time that can be neutral (*s* = 0, green cell) or increase self-renewal bias (*s* > 0, brown cell), leading to the formation of genetic clones. Artwork includes images by Servier Medical Art licensed under CC BY 3.0. **b**, VAF measurement *v*(*t*_0_) at initial timepoint *t*_0_ versus gradient in VAF, $(v(t_{end}) - v(t_0))/(t_{end} - t_0)$, between initial and last timepoints *t*_0_ and *t*_*end*_ of all variants detected in the LBCs with at least two timepoints. Each data point corresponds to a trajectory in the LBCs and has been colored according to its CHIP status based on the 2% VAF threshold (red box). Blue and orange, respectively, denote whether trajectories achieved a VAF > 2% during the observed time span or not. Note: VAF is displayed on a logarithmic scale, as most mutations are concentrated at low VAF. **c**, Number of trajectories passing the currently used 2% VAF threshold, broken down into whether VAF is increasing or decreasing from the first to last timepoint. **d**, Fitness effects of mutations grouped by gene and ranked by median fitness. The posterior probability distribution of the fitness as inferred from our model of clonal dynamics is displayed for each mutation (only the 90% interval is shown). The sample size, *n*, of observed variants in each gene is denoted in brackets. When more than one mutation is observed in a gene, we further display a box plot showing the median and exclusive interquartile range of the MAP fitness estimates associated with the gene.

Stem cell fitness is defined as the proliferative advantage over cells carrying no or only neutral mutations. It remains incompletely understood to what extent fitness is gene-specific or variant-specific or determined by the bone marrow microenvironment and clonal composition. Earlier estimates suggested a wide spread of fitness effects even for variants of the same gene^14^, which would make it difficult to clinically stratify individuals with CHIP. To determine the fitness effects of the variants identified in our cohorts (Fig. 1a and Extended Data Fig. 1e), we initially selected all CHIP variants in our data using the commonly used criterion of defining any variants with VAF > 2% as CHIP^8,13^ and retaining only those variants with at least two timepoints (Fig. 2b). This approach identified 76 CHIP mutations overall (Fig. 2c). To estimate the fitness effect that each variant confers, we used Bayesian inference and birth–death models of clonal dynamics (Fig. 2a), including all trajectories with at least two timepoints (Supplementary Table 3). The resulting fitness values show an overall dependence of fitness on the gene level (Fig. 2d), with a wide distribution of fitness for some genes, such as *TET2* and *DNMT3A*, but not others, such as *JAK2* (which are all the same variant).

### Longitudinal trajectories accurately stratify CHIP variants

Because longitudinal data allow direct quantification of the growth in VAF over time, we can inspect the gradients (fluctuations) in VAF for variants that were classified as CHIP based on thresholding. We found that a VAF > 2% threshold not only misses fast-growing and potentially harmful variants (Fig. 2b) but can also include variants whose frequencies are shrinking (Fig. 2b,c) and, thus, either do not confer a fitness advantage or are being outcompeted by other clones. Overall, 70% of CHIP mutations detected by thresholding at 2% VAF were growing during the observed time span (Fig. 2b,c). Longitudinal data, thus, reveal limitations in defining CHIP mutations based on a widely used VAF threshold.

**Fig. 3:**
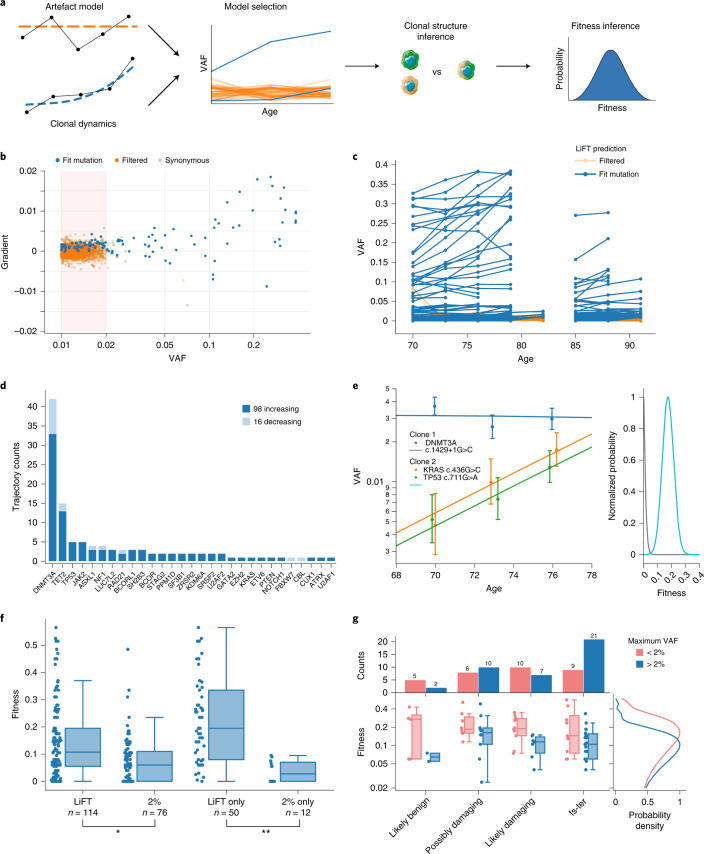
LiFT allows classification of fit variants <2% VAF. **a**, Schematic of LiFT algorithm. LiFT compares a model of clonal dynamics (Fig. 1a) with an artifact model and performs Bayesian model selection. The subsequent steps to infer clonal structure and fitness distributions are as in Fig. 1a. **b**, Gradient in VAF versus VAF for variants detected in the LBCs with at least two timepoints and at least one VAF > 1% per trajectory, with filtered (orange), fit (blue) and synonymous (light green dots) mutations, classified by LiFT on a logarithmic scale. **c**, Longitudinal trajectories of fit (blue) and filtered (orange) mutations linked to age in years. **d**, Number of trajectories classified as fit by LiFT, broken down into increasing or decreasing VAF from first to last timepoint. **e**, Left, deterministic fit of all mutations selected by LiFT in an individual of the LBC cohorts using the inferred optimal clonal structure (Supplementary Information Methods, Appendix B). 90% CIs associated with binomial sampling noise are shown for each data point. VAF is displayed on a logarithmic scale. Right, posterior distribution of fitness associated to each clonal structure. **f**, Fitness effects of variants broken down by filtering method. The sample size, *n*, and statistical analyses comparing the distribution of fitness, computed using the non-parametric Kruskal–Wallis test, are highlighted (*H = 14, *P* = 1 × 10^−4^; **H = 18, *P* = 1 × 10^−5^). **g**, Fitness of variants selected as fit by LiFT broken down by their maximum VAF, >2% and <2%, and damage prediction. The top row displays a bar plot of variant counts for each category. The bottom row displays box plots showing the median and interquartile range of the distribution of MAP fitnesses by damaging prediction displayed on a logarithmic scale to emphasize relative differences in fitness between variants. Consequently, of a total of 89 variants with a damage prediction, 17 variants with fitness below 2% are not shown but are reported in Supplementary Tables 4–6. A marginal plot shows the Gaussian kernel density estimation of the MAP fitness values. fs, frameshifts; ter, terminations.

**Extended Data Fig. 2:**
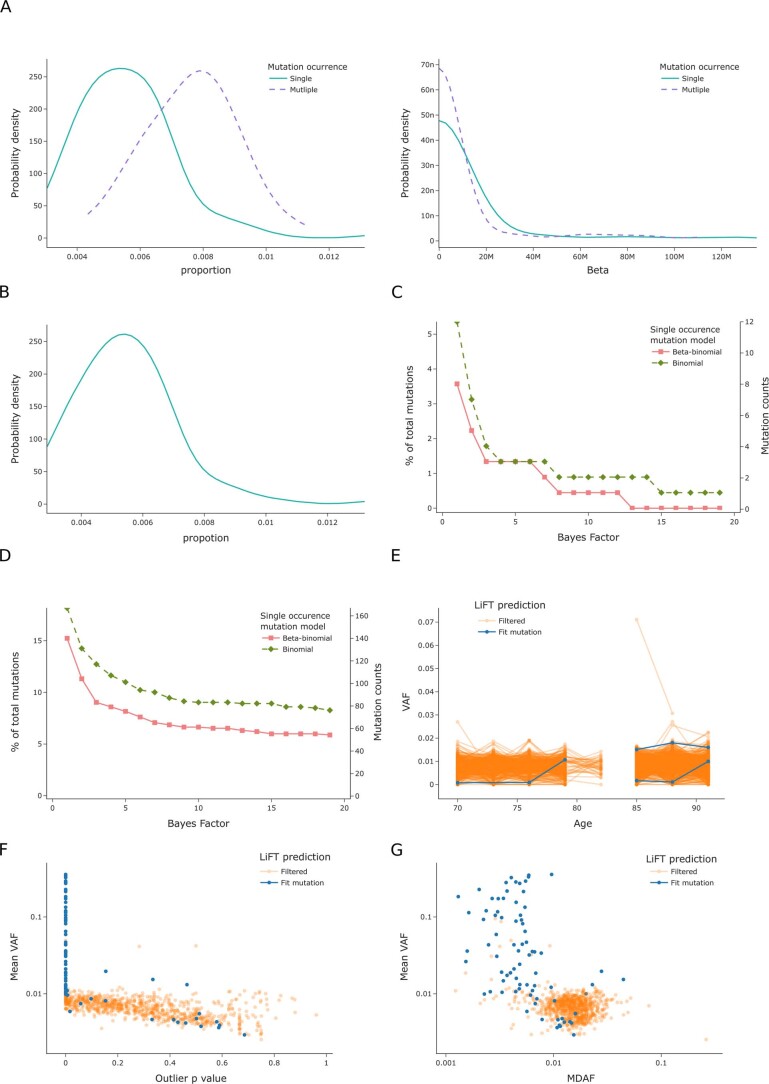
LiFT Method Details. **a**. Prior distributions for the beta-binomial model for sequencing artefacts. Priors are constructed separately for mutations with a single occurrence and mutations with multiple observations in the LBCs (see SI methods Section 2.3). **b**. Prior distribution of the proportion for the binomial model for sequencing artefacts. This prior is constructed only for mutations with a single occurrence in the LBC. **c**. Effect of the Bayes Factor (BF) threshold on the number of non-synonymous variants selected as fit using LiFT. In red, we show the results assuming that sequencing artefacts always follow a beta-binomial model, regardless of the mutation occurrence in the LBC. In green, we show the results where the sequencing artefact model assumes a binomial model for single occurring mutations and a beta-binomial model for mutations with multiple occurrences in the LBCs. **d**. Effect of the BF on the number of synonymous variants selected as fit using LiFT. Colour coding as in Fig. S2C. **e**. Longitudinal trajectories of non-synonymous variants coloured by their LiFT status; fit (blue) and filtered (orange). **f**. Comparison between LiFT status and the VAF Outlier P-value. Each data point corresponds to a trajectory in the LBC and has been coloured according to its LiFT status; fit (blue) and filtered (orange). The coordinates of each data point are given by the average VAF Outlier p-Value and their average VAF. **g**. Comparison between LiFT status and the Minimal Detectable Allele Fraction (MDAF). Each data point corresponds to a trajectory in the LBC and has been coloured according to its LiFT status; fit (blue) and filtered (orange). The coordinates of each data point are given by the average MDAF and their average VAF. Note that the MDAF is shown on a logarithmic scale.

To overcome the limitations of a threshold-based selection of fit variants, we sought to filter variants based on longitudinal information, by comparing a stochastic model of clonal dynamics with a model of sequencing artifacts (Fig. 3a). This novel approach, which we named LiFT, allows classification of fit variants even for VAF < 2%. LiFT classification of fit variants broadly agreed with noise profile statistics from the ArcherDX pipeline (Extended Data Fig. 2f,g) but identified additional variants by leveraging the longitudinal nature of the data. LiFT classification resulted in 114 variant trajectories (Fig. 3b–d and Extended Data Fig. 2a–g), 86% of which grew over the observed time span. We note that the VAF of fit mutations may still shrink over time due to the presence of an even fitter clone in the same individual. This is in contrast to thresholding at 2% VAF, with only 70% of variants identified to be growing and, thus, likely to confer a fitness advantage. Of the 114 variants we detected, 50 would not have been detected using the previous VAF threshold filter. We, therefore, recomputed fitness estimates for this new set of fit trajectories (Fig. 3e,f). Growing variants that were missed by the traditional filtering method include highly fit variants such as *U2AF1* Q157R (fitness 33.5%) and *DNMT3A* R882H (fitness 16%) (Fig. 3c,g and Supplementary Table 4). VAF thresholding did not identify any *TP53* variants. However, LiFT identified four *TP53* mutations, all of which were growing over the observed time course (Fig. 3c,g and Supplementary Table 4). In addition, all of those were either termination/frameshift mutations or previously reported as cancer-associated in the Catalogue of Somatic Mutations in Cancer (COSMIC)^20^ and classified as likely damaging (Supplementary Table 5). Moreover, all *TP53* variants led to high fitness effects; thus, our filtering method allows us to identify potentially harmful variants at very low VAFs. Overall, the variants detected by LiFT were of higher fitness than those detected by VAF thresholding (Fig. 3f; Kruskal–Wallis H = 14, *P* = 1 × 10^−4^), with an even larger effect size when comparing variants that are exclusive to each filtering algorithm (Fig. 3f; Kruskal–Wallis H = 18, *P* = 1 × 10^−5^).

We further stratified variants using seven computational predictors recently identified as being most useful for identifying pathogenic mutations^21–27^ (Fig. 3g and Supplementary Table 5), categorizing the most prevalent CHIP variants into likely damaging (21 variants), possibly damaging (20 variants) and likely benign (11 variants) as well as frameshifts and terminations (37 variants, which are also most likely damaging to protein structure and, thus, protein function; Supplementary Table 6). Our novel LiFT algorithm, therefore, produces a low false discovery rate of pathogenic variants, with 88% of the detected fit variants being predicted to be possibly damaging, frameshift or termination.

Taken together, applying a probabilistic model of clonal dynamics to longitudinal sequencing data results in a novel method—the LiFT algorithm—that improves on the threshold-based definition of CHIP mutations (Fig. 3a). The LiFT algorithm replaces an arbitrary cutoff on VAF by a choice of false discovery rate (through a Bayes factor threshold) and, as a result, selects fewer trajectories with shrinking VAF (Figs. 2b,c and 3b–d).

### Clinical relevance of LiFT

**Fig. 4:**
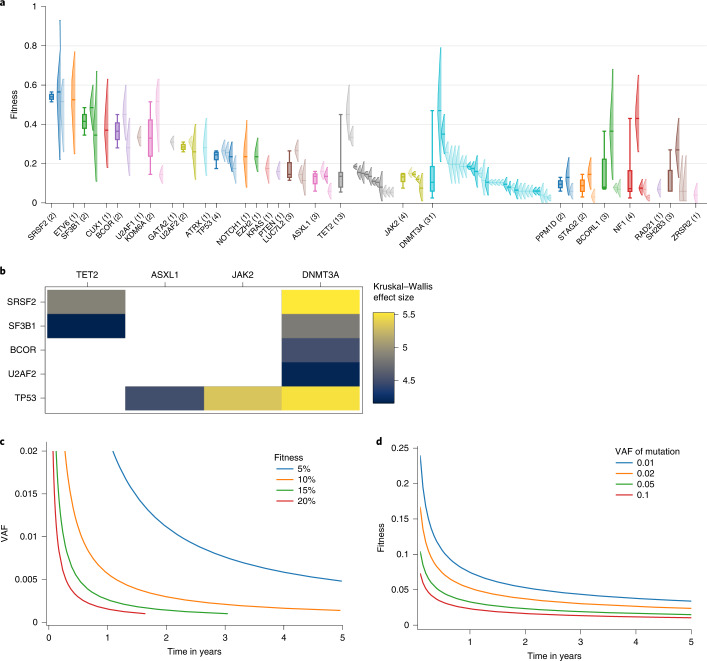
Clinical relevance of LiFT. **a**, Fitness effects of mutations selected as fit by the LiFT algorithm, grouped by gene and ranked by median fitness. The posterior probability distribution of the fitness as inferred from our model of clonal dynamics is displayed for each mutation (only the 90% interval is shown). The sample size, *n*, of observed variants in each gene is denoted in brackets. When more than one mutation is observed in a gene, we further display a box plot showing the median and exclusive interquartile range of the MAP estimates of fitness associated with the gene. **b**, Analysis of variance of the distribution of fitness across genes. Heat map of all statistically significant (*P* < 0.05) Kruskal–Wallis H statistics, labeled by effect size, computed for all combinations of pairs of genes. The effect size is only shown for statistically significant relations. Variants with a fitness below 2% were left out of this study, as our prediction classifies them as conferring no or a negligible fitness advantage. **c**, Minimum referral time in years based on 2 standard deviations below the expected growth of a clone given an initial VAF and fitness. Each line shows the initial size of mutation versus referral time for a given fitness. **d**, Minimum detectable fitness at referral observation based on 2 standard deviations below the expected growth of a clone given an initial VAF and fitness. Each line shows minimum detectable fitness versus referral time for an initial clone size.

**Extended Data Fig. 3:**
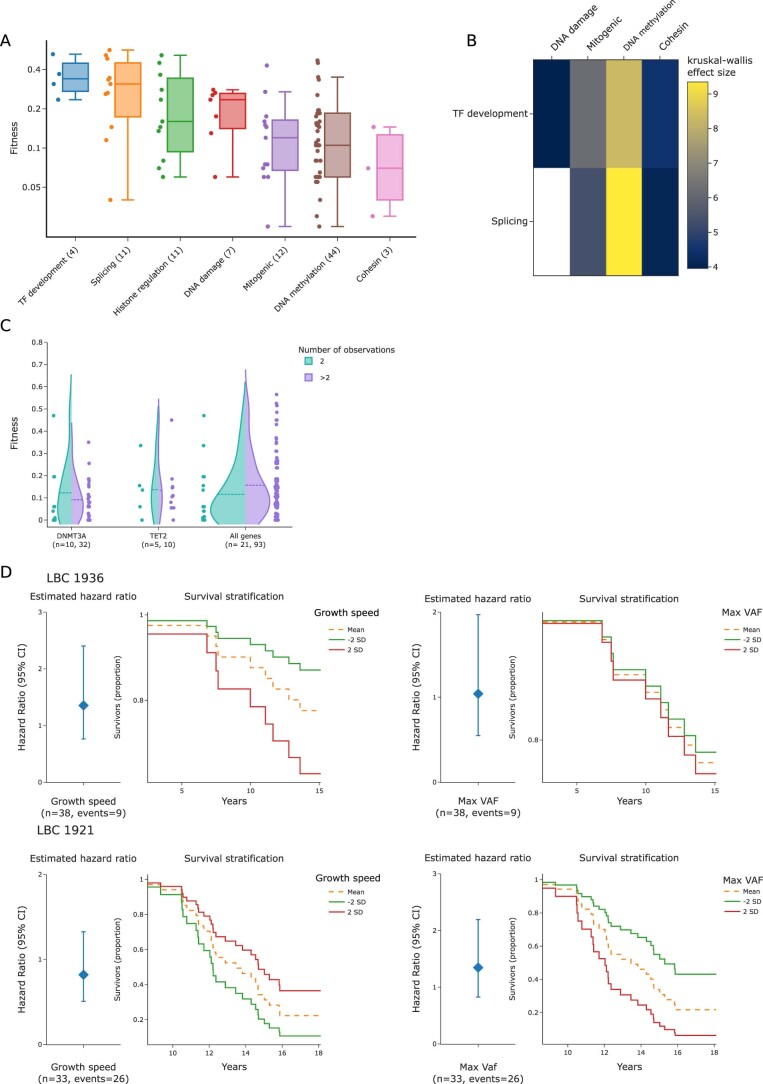
Clinical Relevance of LiFT - Supporting Material. **a**. Distribution of fitness by gene category. Genes are grouped according to their biological function into DNA methylation (*TET2, DNMT3A*), Splicing (*SF3B1, U2AF1, SRSF2, U2AF2, ZRSR2, LUC7L2, DDX41*), mitogenic function (*KRAS, NF1, JAK2, JAK3, SH2B3, PTEN, PTPN11, NRAS*), cohesin (*RAD21, STAG2*), DNA damage (*TP53, CDKN2A, PPM1D, ATRX*) and Transcription factors (TF) important during development (*GATA2, RUNX1, NOTCH1, CUX1, ETV6*). The sample size, n, of each gene category is denoted in brackets. For each gene category we display a boxplot showing the maximum a posteriori (MAP) estimates of fitness for variants in the category, as well as the median and exclusive interquartile range. **b**. Analysis of variance of the maximum posterior fitness estimates across gene categories. Heatmap of all statistically significant (p < 0.05) Kruskal-Wallis H statistics, labelled by effect size, computed for all combinations of pairs of genes. The effect size is only shown for statistically significant relations. Variants with a fitness below 2% were left out of this study as our prediction classifies them as conferring no or a negligible fitness advantage. **c**. Influence of the number of time-points in a trajectory on the inferred fitness distributions. We show the maximum posterior estimates for genes *DNMT3A* and *TET2* and for all LiFT variants split according to the number of time-points. **d**. Survival analysis (Cox proportional hazards regression model) broken down by cohort and covariates. LBC1921 and LBC1936 are analysed separately given their difference in age during the observed time-span. (left) Error bar showing the inferred hazard ratio coefficient and 95% CI for each regression study, as well as the sample size, n, and the number of observed events in each analysis. Note that none of the survival analyses shown are statistically significant. The complete summary for each analysis is found in Supplementary Table 7. (right) Kaplan-Meyer survival plots for the LBC cohort stratified using 2 standard deviations of the analysed covariate.

We further analyzed differences in the distributions of fitness between genes using a non-parametric test. Despite having small sample sizes for many genes, we still detected statistically significant differences among the distributions of fitness effects (Fig. 4a,b). In particular, we found that mutations in *TP53*, *SF3B1* and *SRSF2* conferred a higher fitness advantage over mutations in commonly mutated CHIP genes, such as *JAK2* and *DNMT3A*. We also tested differences in fitness by genes when summarized into functional categories and found trajectories of genes involved in DNA methylation to have lower fitness than genes involved in splicing and genes for transcription factors that are relevant in development (Extended Data Fig. 3a,b).

Differences in the distribution of fitness allow us to predict the future growth of mutations from initial timepoints. For example, if a patient presents with a variant in a gene with 10% fitness at 1% VAF, its growth could be confidently measured after 7 months (Fig. 4c), warranting a clinical follow-up over that timeframe to confirm or revise the fitness estimate. Conversely, the time between observations places a lower bound on the fitness that can be measured for mutations of a given VAF (Fig. 4d). These data can then inform on the timeframe for close clinical monitoring and early detection of disease.

Ableson et al.^16^ compared CHIP carriers who never developed acute myeloid leukemia (AML) with CHIP where individuals subsequently developed AML, and they found that the number of mutations, the mutational burden and the size of the larger driver clone were associated with the risk of progression to AML. In the present study, we carried out a survival analysis to correlate the maximum observed VAF of mutations and survival. This correlation was stronger in the older cohort (LBC1921) although not statistically significant (hazard ratio (HR) = 1.35; 95% confidence interval (CI) 0.83, 2.19; *P* = 0.23) due to the small sample size (Extended Data Fig. 3d and Supplementary Table 7). In the younger cohort (LBC1936), we found that survival better correlated with the speed of growth of a mutation, although this was, again, not statistically significant (HR = 1.35; 95% CI 0.76, 2.4; *P* = 0.3) (Extended Data Fig. 3d and Supplementary Table 7).

Notably, only two timepoints are necessary to apply LiFT, making this a widely applicable method for existing cohorts and future studies (Extended Data Fig. 3c). We propose the use of LiFT over thresholding for clinical practice.

## Discussion

The clinical potential for stratifying progression of CHIP depends on whether genes confer distinct fitness advantages. Indeed, most studies so far have not shown a clear distinction of fitness effects on a gene basis and have shown considerable overlap in fitness coefficients among variants of different genes. We show that fitness can substantially differ by gene and gene category. Combining longitudinal data with a new method to identify CHIP variants allows for more accurate fitness estimates of CHIP than cross-sectional cohort data and motivates further studies with increased sample sizes.

**Extended Data Fig. 4:**
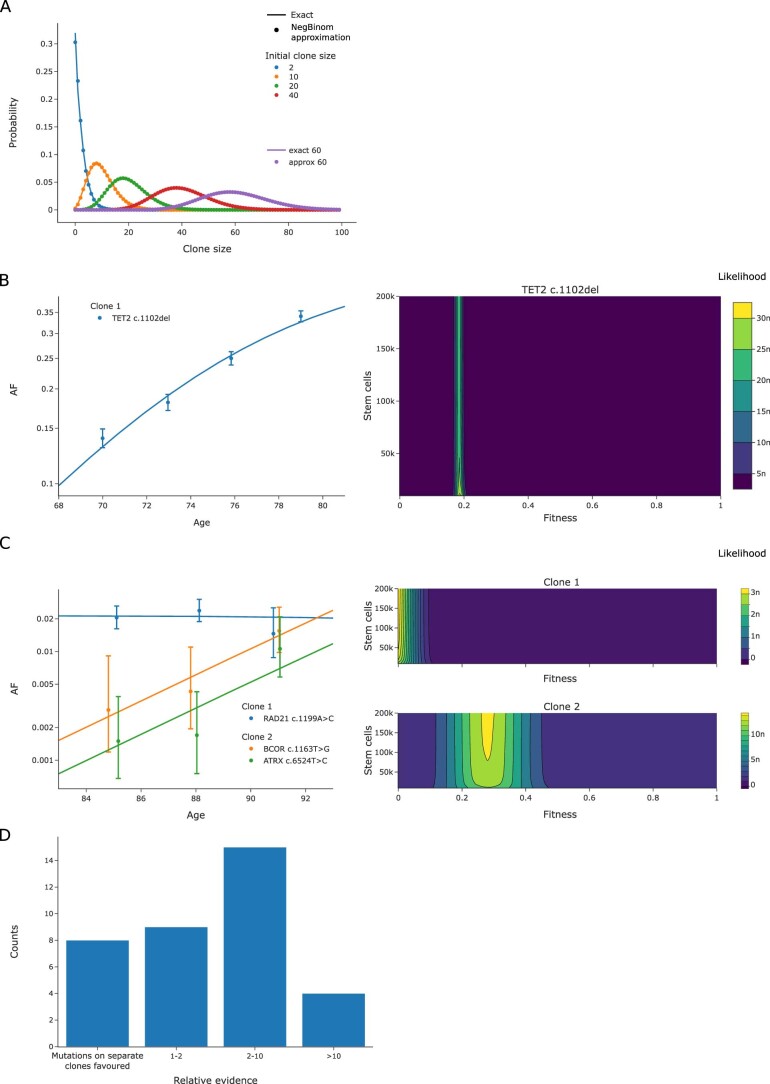
Clonal Dynamics and Inference - Supporting Material. . **a**. Approximation of a neutral birth-death model using the negative binomial distribution. The exact model assumes symmetric divisions occur every 40 weeks, or 1.3 divisions per year, and has no bias towards self-renewal (see SI methods Section 1). **b**. Deterministic trajectory (see SI methods Appendix B) with maximum a posteriori (MAP) fitness and fitted time of mutation (left) and joint posterior distribution of fitness and number of wild-type HSPCs population (right) inferred from an individual with a single mutation selected by LiFT. 90% confidence intervals associated with binomial sampling noise are shown for each data point. Note that VAF is displayed on a logarithmic scale to highlight relative differences and the initial exponential growth of clones. Also note that a small random horizontal jitter has been added to data points to avoid overlapping of confidence intervals. **c**. Deterministic trajectory (see SI methods Appendix B) with maximum posterior fitness and fitted time of mutation (left) and joint posterior distribution of fitnesses and number of wild-type HSPCs inferred from an individual with three mutations, selected by LiFT, occurring in two clones. 90% confidence intervals associated with binomial sampling noise are shown for each data point. Note that VAF is displayed on a logarithmic scale to highlight relative differences and the initial exponential growth of clones. Also note that a small random horizontal jitter has been added to data points to avoid overlapping of confidence intervals. **d**. Evidence supporting the clonal structure selected by our Bayesian model comparison relative to the model assuming no mutations co-occur on the same clone. The evidence is only shown for non-trivial cases where more than one mutation was selected by LiFT in an individual.

Our fitness estimates are independent of the time when the mutation was acquired. In cross-sectional studies, fitness estimates are generally (inversely) correlated with the mutation rate, introducing additional uncertainty^14^. In contrast, our fitness estimates are based on the observed growth among longitudinal samples and, thus, also take into account other mutations in an individual. The resulting fitness estimates are largely independent of hematopoietic stem cell absolute numbers (Extended Data Fig. 4b,c).

The strength of our approach, combining longitudinal data with our LiFT algorithm, is exemplified by *U2AF1* and *TP53*, for which no variants were identified by a 2% VAF threshold (Fig. 2b,c). In contrast, our LiFT method identified one *U2AF1* and four *TP53* variants, all of which are conferring a fitness advantage, scored as possibly damaging in our missense variant effect analysis and have been previously reported in COSMIC^20^ (Fig. 3g and Supplementary Tables 4 and 5). Moreover, we pick up the *DNMT3A R88H* variant with LiFT but not with 2% VAF thresholding—a mutation that is well-reported in the context of leukemia^28^. Therefore, for patients with those variants, close clinical monitoring for early detection of disease such as leukemia is merited.

**Extended Data Fig. 5:**
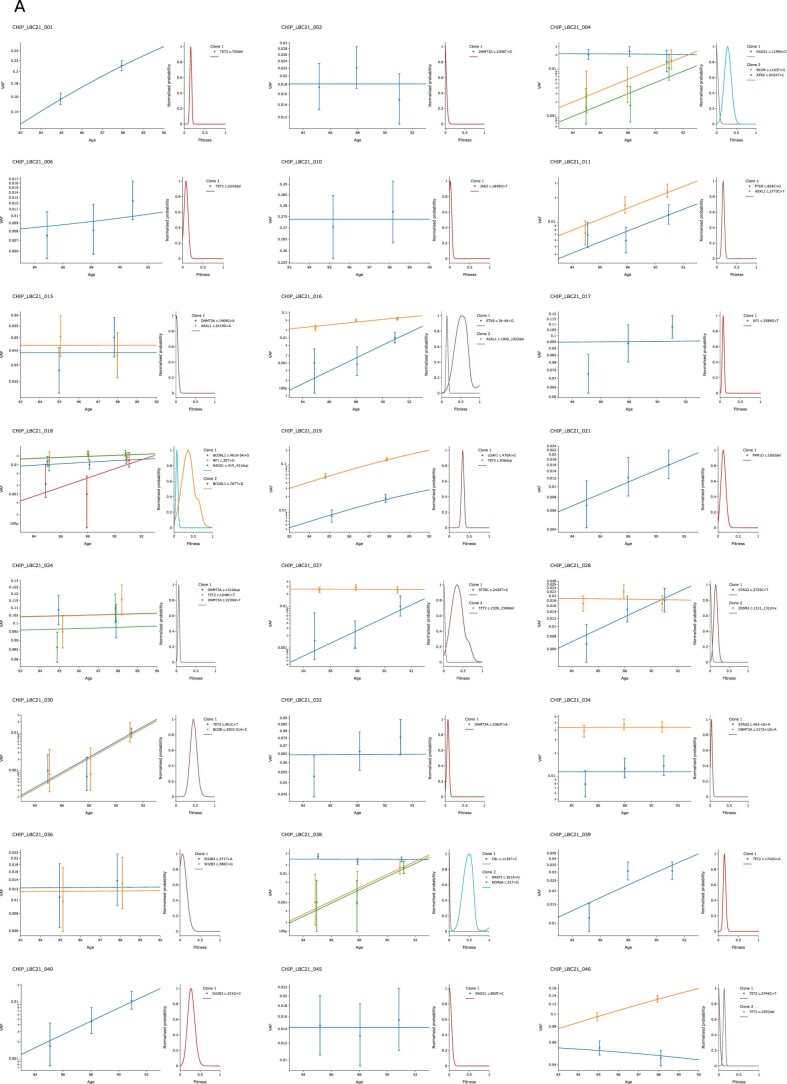
Deterministic Visualisation of Mutational Trajectories in the LBC21. a. Deterministic trajectories (see SI methods Appendix B) with maximum a posteriori (MAP) fitness and wild-type stem cells and fitted time of mutation (left) and posterior distribution of fitness associated to each clonal structure (right) inferred for all mutations selected by LiFT in each participant of the LBC1921 cohort. 90% confidence intervals associated with binomial sampling noise are shown for each data point. Note that VAF is displayed on a logarithmic scale to highlight relative differences and the initial exponential growth of clones. To use a logarithmic axis, data points with zero observations have been replaced by VAF = 0.001, or a factor of 10 below our observation threshold. Also note that a small random horizontal jitter has been added to data points to avoid overlapping of confidence intervals.

**Extended Data Fig. 6:**
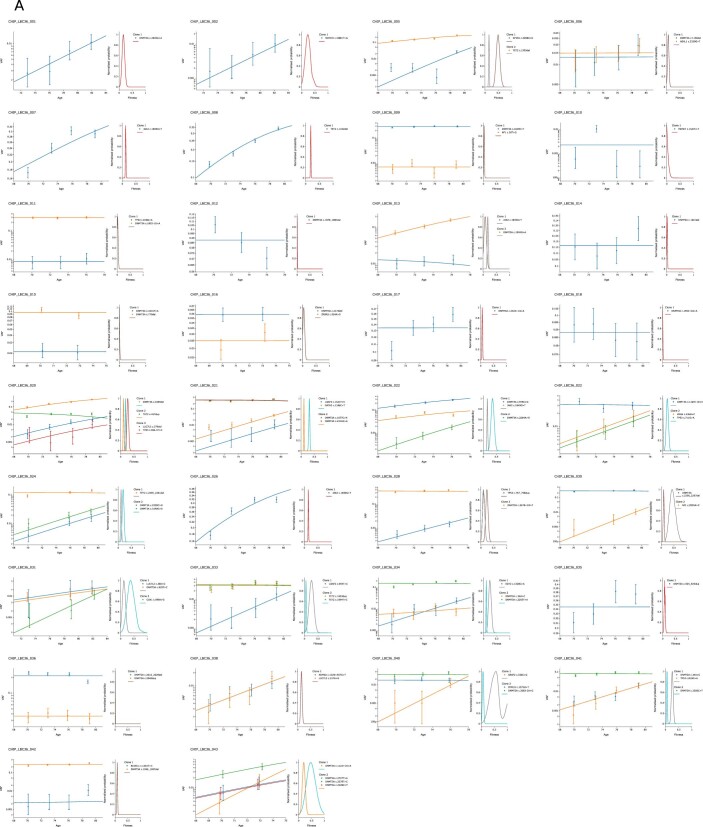
Deterministic Visualisation of Mutational Trajectories in the LBC36. a. Deterministic trajectories (see SI methods Appendix B) with maximum a posteriori (MAP) fitness and wild-type stem cells and fitted time of mutation (left) and posterior distribution of fitness associated to each clonal structure (right) inferred for all mutations selected by LiFT in each participant of the LBC1936 cohort. 90% confidence intervals associated with binomial sampling noise are shown for each data point. Note that VAF is displayed on a logarithmic scale to highlight relative differences and the initial exponential growth of clones. To use a logarithmic axis, data points with zero observations have been replaced by VAF = 0.001, or a factor of 10 below our observation threshold. Also note that a small random horizontal jitter has been added to data points to avoid overlapping of confidence intervals.

Combining longitudinal data with LiFT enables a personalized approach managing CHIP (Extended Data Figs. 5 and 6). Longitudinal data allow quantifying fitness effects even for mutations not seen in large cohorts, as cross-sectional fitness estimation requires a mutation to be observed in multiple individuals. Our method offers clinicians a way forward for patient stratification even for unique variants occurring in single individuals, because two timepoints for one individual suffice to estimate fitness, including uncertainty quantification (Fig. 4e). We have provided a prediction of the time required between first and second observations to be able to accurately infer fitness, depending on the initial VAF of a mutation in an individual (Fig. 4c). For high fitness mutations (>10%), a follow-up clinical observation could be performed after only a few months, even for small clones (1% VAF or less). Conversely, the time between observations places a lower bound on the fitness that can be measured for mutations of a given VAF (Fig. 4d). In the future, these data can be used to inform time to the next appointment for close clinical monitoring of patients with clones containing highly fit variants, which will likely outcompete other clones. Using longitudinal data to better quantify and predict clonal progression in our study, however, comes with a tradeoff in the lower number of participants in our cohort and limits the power of cross-sectional analysis to find associations.

In addition, our inference method aims to resolve the clonal composition of multiple mutations in an individual. Specifically, we can now infer the likely co-occurrence of mutations from longitudinal data. Current cross-sectional studies do not take into account the clonal composition of individuals and, therefore, make predictions of the isolated effect of a mutation. In contrast, we are able to link fitness to clones carrying a specific combination of mutations that is unique to each individual, without relying on any prior knowledge of variant-specific fitness effects (Supplementary Table 4).

## Methods

### Participant samples and ethics

This study complies with all relevant ethical regulations. The study protocol was approved by NHS Lothian (formerly Lothian Health). Informed consent was given by all participants. Ethics permission for LBC1936 was obtained from the Multi-Centre Research Ethics Committee for Scotland (wave 1: MREC/01/0/56), the Lothian Research Ethics Committee (wave 1: LREC/2003/2/29) and the Scotland A Research Ethics Committee (waves 2, 3, 4 and 5: 07/MRE00/58). Ethics permission for LBC1921 was obtained from the Lothian Research Ethics Committee (wave 1: LREC/1998/4/183; wave 2: LREC/2003/7/23; wave 3: 1702/98/4/183) and the Scotland A Research Ethics Committee (waves 4 and 5: 10/MRE00/87).

LBC1921 contains a total of 550 participants at wave 1 of their testing (performed between 1999 and 2001) with a gender ratio of 234:316 (male:female) and a mean age at wave 1 of 79.1 years (s.d. = 0.6) (Supplementary Table 1)^17^. LBC1936 contains a total of 1,091 participants at wave 1 of their testing (performed between 2004 and 2007) with a gender ratio of 548:543 (male:female) and a mean age at wave 1 of 69.5 years (s.d. = 0.8) (Supplementary Table 1)^17^. We previously identified 73 participants with CHIP at wave 1 (ref. ^18^). We sequenced DNA from those 73 LBC participants using a targeted gene panel (Supplementary Table 8) and added 16 LBC participants with previously unidentified CHIP and 4–5 timepoints. We have accepted 85 of 89 participants for inclusion in our study, removing four participants for failing to meet quality criteria (low library complexity), with a total of 248 samples together with 14 ‘Genome in a Bottle’ (GIAB) controls, two per sequencing batch (Supplementary Table 9)^29^. In addition, two individuals carrying the *JAK2V617F* mutation received treatment for leukemia after the first respective timepoint available, potentially driving the observed reductions in clone size. Those patients were omitted from further analysis after sequencing (Fig. 1h).

### Targeted, error-corrected sequencing and data filtering

DNA was extracted from Ethylenediaminetetraacetic acid (EDTA) whole blood using the Nucleon BACC3 kit (Sigma-Aldrich, GERPN8512), following the manufacturer’s instructions. Libraries were prepared from 200 ng of each DNA sample using the Archer VariantPlex® 75 Myeloid gene panel and VariantPlex® Somatic Protocol for Illumina sequencing (Invitae, AB0108, and VariantPlex®-HGC Myeloid Kit for Illumina; Supplementary Table 9), including modifications for detecting low allele frequencies. Sequencing of each pool was performed using the NextSeq 500/550 High-Output version 2.5 (300 cycle) kit on the NextSeq 550 platform (Illumina). To inform reproducibility, background model for error and batch correction, we sequenced two GIAB DNA samples in each batch of samples (DNA NA12878, Coriell Institute)^29^.

Reads were filtered for phred ≥30 and adapters removed using Trimmomatic (version 0.27)^30^ before undergoing guided alignment to human genome assembly hg19 using bwa-mem (version 0.7.17)^31^ and bowtie2 (version 2.2.1)^32^. Unique molecular barcodes (ligated before PCR amplification) were used for read de-duplication to support quantitative multiplexed analysis and confident mutation detection. Within targeted regions, variants were called using three tools (Lofreq (version 2.1.0)^33^, Freebayes^34^ and Vision (ArcherDX version 6.2.7, unpublished)), building a consensus from the output of all callers (Supplementary Table 2).

All filtered variants at 2% VAF met the following criteria: (1) the number of reads supporting the alternative allele surpasses the coverage criteria while exhibiting no directional biases (AO ≥ 5, UAO ≥ 3); (2) variants are significantly underrepresented in the Genome Aggregation Database (gnomAD; *P* ≤ 0.05)^35^; (3) variants are not obviously germline variants (stable VAF across all waves ~0.5 or ~1) that may have been underrepresented in the gnomAD due to the narrow geographical origin of the LBC participants; and (4) contain events that are overrepresented across the dataset—generally frameshift duplications and deletions—whose reads share some sequence homology to target regions yet are likely misaligned artifact from the capture method (Supplementary Table 2). In addition, we manually curated this list, checking for variants that were previously reported, as per Jaiswal et al.^5^, in COSMIC^20^ or in the published literature (Supplementary Table 10). Finally, for any variant that surpassed the above criteria at VAF ≥ 2% across the measured time period, we included other participant-matched data points regardless of VAF level (Extended Data Fig. 1a,b).

To further mitigate against the diverse sources of noise that can occur in any sequencing experiment, which can become especially problematic when attempting to detect variants at low VAFs, the ArcherDX variant-calling platform leverages the pan-dataset coverage levels of each sample and the GIAB controls to establish a position-specific noise profile and, thus, ascertain the limit of detection (LOD) for each variant discovered in our panel. Here, we report two parameters for each variant: (1) the minimal detectable allele fraction (95% MDAF; Extended Data Fig. 1c), which describes the minimum VAF that a variant can be detected in our data, in essence describing the LOD for each event; and (2) the VAF outlier *P* value, which denotes the probability that any variant call could have been generated by sequencing noise given the position-specific noise distribution across our GIAB controls and the pan-dataset coverage levels of our samples, thus allowing us to discern overrepresented sequencing artifacts from real events (Extended Data Fig. 1d).

### Computational prediction of missense variant effects

To predict which missense variants are most likely to be damaging, we used seven computational variant effect predictors recently identified as being most useful for identifying pathogenic mutations^21–27^. Specifically, for each variant identified in this study, we determined what fraction of previously identified pathogenic and likely pathogenic missense variants from ClinVar and what fraction of variants observed in the human population from gnomAD version 2.1 for each computational predictor. We then averaged these fractions across all predictors. Note that DeepSequence^26^ was not run for all proteins due to its computational intensiveness and difficulty of running on long protein sequences. We also performed predictions of missense variant (de)stabilization using FoldX 5.0, using the experimentally determined protein structure, if available, and the AlphaFold model^36,37^.

### Mathematical model of clonal dynamics to infer fitness

Given the longitudinal nature of this study, we can use the probabilistic solution of an established minimal model of cell division^14,38^ to infer the parameter distribution resulting in the observed time evolution of VAF trajectories in a participant’s genetic profile (Fig. 2a). For each individual, we simultaneously estimated the fitness of variants as well as the size of the stem cell pool, without needing to estimate the time of mutation acquisition.

In this model, cells exist in two states: stem cells (SCs) or differentiated cells (DCs). Under the assumption that DCs cannot revert to a SC state, differentiation inevitably leads to cell death and is treated as such. Furthermore, assuming that each SC produces the same amount of fully differentiated blood cells allows a direct comparison between the VAF of a variant as observed in blood samples and the number of SCs forming the genetic clone (clone size). For an individual with a collection of clones $\{ c_i\} _{i \in I}$, the VAF evolution in time *v*_*i*_(*t*) of a clone *c*_*i*_ corresponds to $v_i(t) = \frac{{n_i(t)}}{{2N(t)}}$, where *v*_*i*_(*t*) is the VAF of the variant at time *t*; *n*_*i*_(*t*) is the number of SCs carrying the variant; and *N*(*t*) corresponds to the total number of diploid HSPCs present in the individual. Finally, we assume that $N(t) = N_w + \mathop {\sum}\nolimits_{i \in I} {n_i(t)}$, where *N*_*w*_ is the average number of wild-type HSPCs in the individual. The bias toward self-renewal of symmetric divisions is parameterized by parameter *s* and determines the fitness advantage of a clone. In normal hematopoiesis, *s* = 0, in which case clones undergo neutral drift. For clones with non-neutral (fitness-increasing) mutations, *s* > 0, and this average clone size grows exponentially in time as $e^{s(t - t_0)}$ from an initial population of one SC at the time of mutation acquisition *t*_0_. The full distribution of clone sizes is well-approximated by a negative binomial distribution matching the mean (exponential growth) and variance of the full stochastic solution (Supplementary Information Methods, section 1, and Extended Data Fig. 4a). Because the model dynamics are Markovian (without memory), once we condition on a previously observed timepoint in a trajectory, the prediction for all future times is independent of *t*_0_. From the predicted clone size distributions, we can infer the marginal posterior distribution of parameter *s* using Bayes’ theorem (Supplementary Information Methods, section 3)^39^. We further take into account the sampling error during sequencing to estimate the distribution of clone sizes at the start and end of each time interval in the longitudinal sequencing data. Here, we approximate this sampling error as binomial.

When multiple fit clones are present in an individual, we constrain the inference to share the SC pool size *N*(*t*) for all variant trajectories in this individual. This increases the data:parameter ratio and produces richer dynamics, where the evolution of exponentially growing clones can be suppressed by the growth of a fitter clone. This implies that even non-competitive models, where trajectories grow independently of each other, will result in competitive dynamics in the observed VAF trajectories as variants strive for dominance of the total production of blood cells.

We take into account possible clonal substructures for all fit variants in an individual, selecting models with co-occurring mutations on the same clone if they are more likely after biasing against models with multiple mutations per clone, as these are presumed to be rarer (Supplementary Information Methods, section 2.4.7). The evidence supporting the optimal clonal structure, determined by Bayesian model comparison, relative to the model assuming no mutations co-occur on the same clone is shown in Extended Data Fig. 4d. We then infer the posterior fitness distributions per clone for the most likely clonal model in every participant.

Once we have inferred the posterior distributions of the parameters, we use the mode of the distribution (maximum a posteriori (MAP) estimate) for each mutation to visualize the deterministic—that is, average—growth curves. These result in the logistic time evolution of its corresponding VAF,

$$v(t) = \frac{1}{{2 + 2N_we^{ - s(t - t_0)}}},$$

where we determine the time of mutation acquisition *t*_0_, which is used only for plotting, using maximum likelihood (Supplementary Information Methods, Appendix B). Although deterministic fits are not a direct reflection of the inference results of our stochastic model, these can be used to visually assess the ‘goodness of fit’ of the fitness MAP estimates and have been included for each participant in LBC1921 and LBC1936, respectively, in Extended Data Figs. 5 and 6.

Note that this model cannot account for loss-of-heterozygosity events.

### LiFT

To select fit variants, we compare the likelihood of the clonal model, including binomial sampling error, to a model of sequencing artifacts. The artifact model assumes that all variability arises from sampling error with a proportion that remains constant over time. For variants that occur more than once in our dataset, we use a beta-binomial model to account for overdispersion, and, for unique variants, we use a binomial model. We select variants as fit only if the model evidence for the clonal model is at least four times that of the artifact model (Supplementary Information Methods, section 2.4, and Extended Data Fig. 2c,d). Fit variants thus selected are taken through to clonal structure model selection and fitness inference as described above.

### Workflow overview

A workflow chart describing the full pipeline and implementation guidance is included in the GitHub repository (see ‘Code availability’). Our pipeline can be applied to other datasets with a few adjustments. Our LiFT algorithm has been tailored to the LBC dataset by extracting parameters from the distribution of synonymous mutation reads, which inform the priors used for our Bayesian inference method (Supplementary Information Methods, section 2.3.3, and Extended Data Fig. 2a–c). Guidance on how to adapt our LiFT algorithm to other datasets is included in the code repository. All other parts of the pipeline, including the extraction of variants using ArcherDx software and the inference of clonal structures and fitness, are directly applicable to other datasets.

### Framework implementation

Both LiFT and Bayesian inference of the posterior distribution of model parameters were implemented in Python version 3.7 (ref. ^40^) with dependencies on Numpy version 1.21.5 (ref. ^41^), Scipy version 1.7.3 (ref. ^42^) and Pandas version 1.3.4. Survival analysis was implemented using Python version 3.7 (ref. ^40^) with dependencies on lifelines version 0.26.4 (ref. ^43^). Data curation was undertaken in Python version 3.7 (ref. ^40^) and R base^44^, with use of the ‘tidyverse’^45^ suite of packages and plotted with ggplot2 (ref. ^46^).

### Reporting summary

Further information on research design is available in the Nature Research Reporting Summary linked to this article.

## Online content

Any methods, additional references, Nature Research reporting summaries, source data, extended data, supplementary information, acknowledgements, peer review information; details of author contributions and competing interests; and statements of data and code availability are available at 10.1038/s41591-022-01883-3.

## Supplementary information

Supplementary Information Methods—Detailing mathematical framework of methodology in three sections with two appendices

Reporting Summary

Tables 1–10 Supplementary Table 1: Cohort information. Supplementary Table 2: Complete list of unique variants detected at 2% VAF, including the maximum clone size (VAF) for events in all participants. Supplementary Table 3: Complete list of unique fit CHIP variants at 2% VAF. Supplementary Table 4: LiFT-Filter variants fitness estimates. Supplementary Table 5: Damage predictions for all single-nucleotide variants. Supplementary Table 6: Variants with a frameshift or termination mutation below 2% VAF. Supplementary Table 7: Survival analysis on the effects of maximum VAF and clone growth speed. Supplementary Table 8: Target genes of the ArcherDX VariantPlex Myeloid Panel. Supplementary Table 9: Sequencing quality control metrics. Supplementary Table 10: Known CHIP mutations.

## Data Availability

We have deposited all data pertinent to this analysis, including the de-identified raw FASTQ read data and processed variant calls for our longitudinal cohort, onto the National Center of Biotechnology Information Gene Expression Omnibus under accession ID GSE178936. LBC phenotypic data are available in the database of Genotypes and Phenotypes (dbGAP) under accession number phs000821.v1.p1. All other Lothian Birth Cohort data are deposited in dbGAP or are provided via the LBC Data Access Collaboration (https://www.ed.ac.uk/lothian-birth-cohorts/data-access-collaboration). Information concerning the cohort is contained here, including its history, data summary tables for both LBC1921 and LBC1936 and data access request forms and contact information to obtain all data points (contact: https://www.ed.ac.uk/profile/simon-cox, simon.cox@ed.ac.uk; timeframe: 1 month to respond).

## Code availability

All code used in this manuscript is available at https://github.com/neilrobertson/LBC_ARCHER.
