# Supplementary material for: Longitudinal dynamics of clonal hematopoiesis identifies gene-specific fitness effects
Source: Nat Med. 2022 Jul 4;28(7):1439–46. doi: 10.1038/s41591-022-01883-3 (PMC9307482; doi:10.1038/s41591-022-01883-3)
Supplement: Supplementary file 1 — Methods—Detailing mathematical framework of methodology in three sections with two appendices [file 41591_2022_1883_MOESM1_ESM.pdf]

nature portfolio

https://doi.org/10.1038/s41591-022-01883-3

Supplementary information

Longitudinal dynamics of clonal hematopoiesis identifies gene-specific fitness effects

In the format provided by the authors and unedited

Supplementary Methods for "Longitudinal dynamics of clonal haematopoiesis identifies gene-specific fitness effects"

Contents

1 Birth-death model of clonal dynamics **3**

2 Filtering longitudinally sampled variants with Bayesian model comparison **4**

2.1 Time series data 4

2.2 Likelihood-based Filter for Time-series data (LiFT) 5

2.3 Sequencing artefact model 6

2.4 Clonal dynamics 8

3 Inferring clonal fitness **13**

Appendices **14**

A A stochastic model of neutral clones **14**

A.1 Evolution of HSPC counts in neutral clones 14

A.2 Distribution of VAF sizes in neutral clones 15

A.3 Maximum size of neutral clones 16

B Deterministic model of VAF evolution **16**

B.1 Maximum likelihood fitting 17

B.2 Visualising stochastic fits using the deterministic model 18

1 Birth-death model of clonal dynamics

Consider a population $X(t)$ of haematopoietic stem and progenitor cells (HSPCs) in an individual at time t. A classical model of stem cell population assumes that HSPCs divide and differentiate according to the following birth-death process, (Till et al., 1964; Watson et al., 2020):

$$\begin{aligned} HSPC &\rightarrow HSPC + HSPC, && \text{with} \quad (\lambda + s)\,\mathrm{d}t, \\ &\rightarrow D + D, && \text{with} \quad \lambda\,\mathrm{d}t, \\ &\rightarrow HSPC + D, && \text{with} \quad A\,\mathrm{d}t, \end{aligned} \tag{1}$$

where D denotes a differentiated cell. Since differentiated cells cannot self-renew, differentiation will result in cell death and is treated as such. The deterministic behaviour of this system is given by

$$\frac{\mathrm{d}X}{\mathrm{d}t}(t) = sX(t). \tag{2}$$

Consequently, the time evolution of the population of HSPCs is

$$X(t) = X(0)e^{st}, \tag{3}$$

where $X(0)$ corresponds to the initial population at time $t = 0$. It is clear that parameter s regulates the excess growth towards self-renewal and dictates the evolution of $X(t)$.

The stochastic behaviour of this model, on the other hand, is more involved. Assuming that $X(0) = 1$, $X(t)$ has the following probability distribution (Kendall, 1948; Bailey, 1990):

$$P\left(X\left(t\right) = k\right) = \begin{cases} \left(1-\alpha\right)\left(1-\beta\right)\beta^{k-1}, & k > 0, \\ \alpha, & k = 0, \end{cases} \tag{4}$$

where

$$\alpha = \frac{\lambda\left(e^{st}-1\right)}{\left(\lambda+s\right)e^{st}-\lambda}, \quad \beta = \frac{\left(\lambda+s\right)\left(e^{st}-1\right)}{\left(\lambda+s\right)e^{st}-\lambda}. \tag{5}$$

More generally, the distribution accounting for the case when the initial population is $a > 0$,

$$p_k^X\left(t, a\right) = P\left(X\left(t\right) = k \mid X\left(0\right) = a\right), \tag{6}$$

can be analytically derived as

$$p_k^X(t,a) = \begin{cases} \sum_{j=0}^{\min(a,k)} \binom{a}{j}\binom{a+k-j-1}{a-1}\alpha^{a-j}\beta^{k-j}(1-\alpha-\beta)^j, & k>0,\\ \alpha^a, & k=0. \end{cases} \tag{7}$$

Of particular relevance is the mean and variance of $X(t)$, if $X(0) = a$:

$$\mu_X(t) = ae^{st} \quad \text{and} \quad \sigma_X^2(t) = \frac{a(2\lambda+s)}{s}e^{st}\left(e^{st}-1\right). \tag{8}$$

Note that the probability distribution, $p_k^X(t,a)$, of clone sizes at time t, conditional on an initial clone size a, looks like a modified negative binomial distribution (7). Thus we can approximate this probability distribution using a negative binomial distribution, $\mathrm{NegBin}(r,p)$ with probability mass function $nb(k;r,p)$, parameterised in terms of the mean μ_X and variance σ_X^2 associated with the birth-death process (8).

That is

$$p_k^X(t,a) \sim nb(k;r,p), \tag{9}$$

with

$$r = \frac{\mu_X^2}{\sigma_X^2 - \mu_X}, \quad \text{and} \quad p = \frac{\sigma_X^2 - \mu_X}{\sigma_X^2}.$$

The negative binomial approximation (9) agrees well with the full expression (7) as shown in SuppFig. 4A.

2 Filtering longitudinally sampled variants with Bayesian model comparison

We here develop a mutation-specific model comparison method to provide evidence of whether sequencing data associated to a genetic mutation is real or the result of a sequencing artefact. We will use Bayesian inference to compare two models, $\mathcal{M}_{seq}$ and $\mathcal{M}_{BD}$, that respectively model the probability of observing a time series data assuming the data was produced by either a sequencing artefact or by a genetic clone following a birth-death process.

2.1 Time series data

The longitudinal nature of the Lothian Birth Cohorts (LBCs) allows us to extract the time series data following the evolution of genetic mutations in blood.

That is each genetic mutation, m, present in an individual, i, is represented by a time series

$$\mathbb{Y}_m^i = \left(Y_j^{i,m} : j \in T \right), \tag{10}$$

where T is the set of time-points and each data point, $Y_j^{i,m} = \left(a_j^{i,m}, d_j^{i,m}, t_j^{i,m} \right)$, is characterised by the read depth and alternate observations of a genetic mutation, $a_j^{i,m}$ and $d_j^{i,m}$ respectively, as well as the time of observation $t_j^{i,m}$. Thus $Y_j^{i,m} \in \mathbb{N}^2 \times \mathbb{R}^+$. We can then denote the time-ordered set of data points by

$$\mathcal{D}_m^i = \left\{ Y_j^{i,m} \right\}_{j=1}^{n_{i,m}} =: Y_{1:n_{i,m}}^{i,m},$$

where $n_{i,m}$ corresponds to the total number of data points in the trajectory. We further group genetic mutations re-occurring in several participants and denote the full set of data associated to a genetic mutation, m, as

$$\mathcal{D}_m = \bigcup_{i \in \mathcal{I}_m} \mathcal{D}_m^i,$$

where the union is taken over all individuals harnessing mutation m, $\mathcal{I}_m$.

2.2 Likelihood-based Filter for Time-series data (LiFT)

Let $\mathcal{D}_m$ bet the set of data associated to a genetic mutation, m, and consider the probability of observing these data under a model $\mathcal{M}$, $P\left(\mathcal{D}_m \mid \mathcal{M}\right)$. Note that

$$P\left(\mathcal{D}_m \mid \mathcal{M}\right) = \prod_{i \in \mathcal{I}_m} P\left(\mathcal{D}_m^i \mid \mathcal{M}\right), \tag{11}$$

since trajectories in different participants are independent.

The likelihood of the model (11) can be expressed in terms of the likelihood of the model parameters as

$$P(\mathcal{D}_m \mid \mathcal{M}_k) = \int_{\theta_k} P\left(\theta_k\right) P\left(\mathcal{D}_m \mid \theta_k, \mathcal{M}_k\right) d\theta_k,$$

where θ_k corresponds to the set of parameters associated to $\mathcal{M}_k$ and $P(\theta_k)$ to their joint prior distribution.

We then compare two models $\mathcal{M}_1$ and $\mathcal{M}_2$ based on the strength of evidence of one model over the other by calculating Bayes factors

$$K_m = \frac{P(\mathcal{M}_1 \mid \mathcal{D}_m)}{P(\mathcal{M}_2 \mid \mathcal{D}_m)} \frac{P(\mathcal{M}_2)}{P(\mathcal{M}_1)} = \frac{P(\mathcal{D}_m \mid \mathcal{M}_1)}{P(\mathcal{D}_m \mid \mathcal{M}_2)}. \tag{12}$$

As described in the main text, we only select $\mathcal{M}_{BD}$ over $\mathcal{M}_{seq}$ if the Bayes Factor is ≥ 4 (SuppFig. 2C, 2D).

2.3 Sequencing artefact model

We first explore model $\mathcal{M}_{seq}$ that assumes the observed data are the result of a sequencing artefact. In what follows we will describe two different models of sequencing artefacts, the binomial and the beta-binomial models, each having different strengths and then justify the use of a hybrid model.

To keep notation simple, below we omit indices i and m, indicating the participant and mutation associated to a time point, $Y_j^{i,m}$, of the LBC data and only reintroduce them when data form multiple trajectories are considered.

Further, since the data arising from sequencing artefacts are distributed independently from each other, the conditional distribution is independent of the previous time point, so that

$$P\left(Y_j \mid Y_{j-1}, \mathcal{M}_{seq}\right) = P(Y_j|\mathcal{M}_{seq}). \tag{13}$$

Since our model for clonal dynamics will be conditional on the first time-point, we will only take into account time-points $t \geq 2$ for the artefact model. This facilitates a fair comparison as the probability of the first time-point would otherwise decrease the artefact model probability.

2.3.1 Binomial model

First we present a simple binomial model of sequencing artefact, $\mathcal{M}_{seq}^{binom}$. Under this model, data are given independently by random variables following a binomial distribution $\mathrm{Binom}(d_j, p)$, with probability mass function $b(a_j; d_j, p)$.

The conditional probability of a single data point Y_j is then given by the probability of seeing a_j alternate observations at a read depth d_j (given p), i.e.,

$$P(Y_j \mid p) = b\left(a_j; d_j, p\right).$$

Combining all individuals $i \in \mathcal{I}_m$ with mutation m, the likelihood of model $\mathcal{M}_{seq}^{binom}$ is

$$P\left(\mathcal{D}_m \mid \mathcal{M}_{seq}\right) = \int_p P(p) \prod_{i \in \mathcal{I}_m} \prod_{j=2}^{n_{i,m}} b\left(a_j^{i,m}; d_j^{i,m}, p\right) dp,$$

where $P\left(p\right)$ is the prior distribution of binomial proportions.

This model also approximately describes the behaviour of neutral mutations (if we assume binomial sampling error as we will do in our clonal dynamics model): mutations without any fitness advantage remain stable in VAF over time, and

for small VAF the fluctuations observed in their longitudinal behaviour are stemming primarily from sampling error.

2.3.2 Beta-binomial model

Next we introduce $\mathcal{M}_{seq}^{beta}$ that assumes data are given by independent random variables following beta-binomial distributions $\mathrm{BetaBin}(d_j^{i,m}, \alpha, \beta)$ with probability mass functions $bb(y; d_j^{i,m}, \alpha, \beta)$. Note that beta-binomial distributions can be interpreted as over-dispersed binomial distributions and have previously been used to capture the behaviour of a wide range of sequencing artefacts (Martincorena et al., 2015). Parameterising $\alpha = \beta p/(1-p)$, for a given $p \in [0, 1]$, the distribution's mean, μ_{BB}, becomes

$$\mu_{BB} = \frac{d_j \alpha}{\alpha + \beta} = d_j p,$$

and models an over-dispersed binomial distribution $B(d_j, p)$.

The conditional probability of a single data point Y_j is then given by the probability of seeing a_j alternate observations at a read depth d_j (given p and β), i.e.,

$$P(Y_j \mid p, \beta) = bb\left(a_j; d_j, \frac{\beta p}{1-p}, \beta\right).$$

Combining all individuals $i \in \mathcal{I}_m$ with mutation m, the likelihood of model $\mathcal{M}_{seq}$ is

$$P\left(\mathcal{D}_m \mid \mathcal{M}_{seq}\right) = \int_p \int_\beta P(p)P(\beta) \prod_{i \in \mathcal{I}_m} \prod_{j=2}^{n_{i,m}} bb\left(a_j^{i,m}; d_j^{i,m}, \frac{\beta p}{1-p}, \beta\right) d\beta dp,$$

where $P(p)$ and $P(\beta)$ are the prior distributions of this model's parameters.

2.3.3 Estimating prior distributions artefact parameters

We infer the prior distributions $P(p)$ and $P(\beta)$ from synonymous mutation data in the LBCs. Synonymous mutations are mutations that do not alter the coded amino acid and are therefore functionally inconsequential. Since these mutations do not confer any fitness advantage they should follow a critical birth-death process and are unlikely to reach VAF $> 10^{-4}$(see Appendix A). The detection limit in our study is VAF > 0.01, therefore any synonymous mutation observed is likely to be either the result of a sequencing artefact or a hitchhiker mutation (neutral mutation co-occurring in a cell with a fitness-inducing mutation). Since hitchhiker mutations are presumably rare (Watson

et al., 2020), we use synonymous mutation data as sequencing artefacts to infer the prior distributions of our artefact model. Note that even if the majority of the data were not artefacts but real mutations, this would not affect our filtering as we aim to distinguish growing fit clones from artefacts or synonymous variants, and in the case of zero fitness the artefact model (which can also approximate neutral fluctuations) will be favoured due to fewer parameters.

Next we detail the process to extract the prior distributions associated with the beta-binomial model. For each synonymous mutation, m, we maximize the likelihood function associated to a sequencing artefact,

$$\mathcal{L}(p, \beta \mid \mathcal{D}_m) = \prod_{i \in \mathcal{I}_m} \prod_{j=2}^{n_{i,m}} bb\left(a_j^{i,m}; d_j^{i,m}, \frac{\beta p}{1-p}, \beta\right),$$

and obtain the collection of optimal parameters

$$\{(p_m, \beta_m)\}_{\mathcal{L}(p_m, \beta_m | \mathcal{D}_m) = max_{p,\beta} \mathcal{L}(p,\beta|\mathcal{D}_m)}.$$

We then use a Gaussian Kernel Density estimator to infer the distribution of optimal parameters p and β that are used as priors for $P(p)$ and $P(\beta)$ (SuppFig. 2A). The prior distribution $P(p)$ (SuppFig. 2B) associated to the binomial model is obtained analogously.

2.3.4 Comparing artefact models with and without overdispersion for unique variants

When faced with synonymous variants occurring only once in the cohort, it is questionable whether a there is any reliable evidence for overdispersion. We therefore compared how many synonymous variants were classified as artefacts by the LiFT algorithm depending on whether we use a binomial or beta-binomial model for such singly occuring variants, while using the beta-binomial model for variants occurring in multiple individuals in both cases. We find that that the choice of error model makes little difference to the synonymous variant classification (3 mutations not classified as artefacts irrespective of the beta-binomial or binomial choice, SuppFig. 2C). For non-synonymous variants, the use of the beta-binomial model for unique variants appears to bias against against detecting fit variants (SuppFig. 2D).

2.4 Clonal dynamics

Here we consider the model $\mathcal{M}_{BD}$ that considers the data to result from true variants in blood cells that are subject to the clonal dynamics of HSPCs, which is modelled as a birth-death process. In the following we will consider a hidden

Markov model, as the true clone sizes X_j at time-point j are inaccessible to us, but relate to the measured Y_j sequencing data through a measurement model (we refer the reader to Ghahramani (2001) for a gentle introduction to hidden Markov models).

2.4.1 Hidden Markov model of clone size evolution

First consider the longitudinal data of a single trajectory $Y^i_{1:n_{i,m}}$ associated to mutation m in participant i, with underlying clone sizes $X^i_{1:n_{i,m}}$. Below we again omit indices i, m to keep the notation simple and only reintroduce them when data form multiple trajectories are considered. Recall from (9) the expression for dynamics of the clone size, $X(t)$, of a genetic clone with fitness $s \geq 0$,

$$BD\left(k, t; k_0, s\right) \coloneqq P\left(X\left(t\right) = k \mid X\left(0\right) = k_0, s\right),$$

for which we will use the short-hand $P(X_j \mid X_{j-1})$ when $t = t_j - t_{j-1}$ is the time between successive measurements Y_{j-1} and Y_j.

However, we do not have direct access to clone sizes, X_j, but only to the alternate allele observations a_j and the read depth d_j, i.e., Y_j. We therefore need to also take into account the observation probability

$$P\left(Y_j = y_j \mid X_j = x_j, N_w\right), \tag{14}$$

for which we will use the short-hand $P(Y_j \mid X_j)$. A detailed derivation of $P(Y_j \mid X_j)$ can be found in 2.4.3, but for now it suffices to note that the observation probability depends not only on the clone size X_j but also on the number of wild-type HSPCs, N_w.

We will now consider the joint probability

$$P(X_{1:n}, Y_{1:n}) = P(X_1)P(Y_1 \mid X_1) \prod_{j=2}^{n} P(X_j \mid X_{j-1})P(Y_t \mid X_t) \tag{15}$$

and marginalize this over the values of the hidden variable, $X_{1:n}$, to get

$$\begin{aligned} P(Y_{1:n} \mid s, N_w) = \sum_{x_1,\ldots,x_n} \frac{1}{A} \overbrace{P(X_1 = x_1)P(Y_1 \mid X_1 = x_1, N_w)}^{\text{initial size uncertainty}} \\ \cdot \prod_{j=2}^{n} \underbrace{P(X_j = x_j \mid X_{j-1} = x_{j-1}, s)}_{\text{clonal growth}} \underbrace{P(Y_t \mid X_t = x_j, N_w)}_{\text{measurement}}, \end{aligned} \tag{16}$$

where we have made explicit the dependence on parameters s, N_w to emphasize that this is a likelihood. A is a normalisation constant to ensure that

$\sum_{x_1} P(X_1)P(Y_1 \mid X_1)/A = 1$, i.e., the first time-point only weights the different paths of clonal dynamics according to the observation probability. We further choose the prior over initial clone sizes $P(X_1)$ to be uniform.

2.4.2 Numerical implementation

The sum over clone sizes, x, in (16) ranges from 0 to ∞, though in practice only a few terms will contribute meaningfully to the probability. For computational efficiency we use 25 samples of x_1 evenly spaced in the 99% interval of the distribution $P(Y_1 \mid X_1)P(X_1)$. At each subsequent time-point ($j \geq 2$) we generate 25 samples of x_j in the 99% interval of $P(Y_j \mid X_j)$, plus a further 25 samples between the 99th percentile of $P(X_j \mid X_q = \max(\{x_q\}))$ and the 1st percentile of $P(X_j \mid X_q = \min(\{x_q\}))$, for $q = 1, \ldots, j-1$, where $\{x_q\}$ are the samples from time-point q (in the 99% interval of $P(Y_q \mid X_q)$). This results in $25 \times j$ samples in total for time-point j. We use these samples to approximate the integrals using the trapezoidal method.

The number of samples used above that are required to generate smooth posterior distributions will depend on the queried resolution in fitness. In Extended Data Figure 6, participant LBC36 038, it can be seen that the sampling described above is insufficient to produce a smooth posterior distributions at a resolution of 201 points in fitness. We found that this could be rectified by increasing the number of samples used to approximate the sum over clone sizes, at the cost of longer computational run-time. This limitation likely relates to the challenge of sampling clone sizes with non-zero probability associated to large VAF observations. To reach VAF 0.5 clone sizes in the current model need to approach infinity (see Section 2.4.3). The range of clone sizes that need to be sampled over thus becomes very large for large VAF. This is a limitation of modelling exponential growth in clone size, which can result in biologically unrealistically large clone sizes. This may be rectified by competitive models where the population of mutated HSPCs does not grow exponentially, but is rather constrained by the biological niche.

2.4.3 Measurement model of sequencing process

We simulate the process of sequencing as a binomial draw $\mathrm{B}\left(d\left(t\right), p\left(X(t), N_w\right)\right)$, with probability mass function $\mathrm{b}(a; d\left(t\right), p\left(X(t), N_w\right))$, where $p\left(k, N_w\right)$ corresponds to the allele frequency of a genetic clone of size x . The allele proportion of a clone of size x is given by $p\left(x, N_w\right) = x/2\left(x + N_w\right)$, where N_w is to the number of wild-type HSPCs. The probability mass function of alternate allele observations for a genetic variant conditional on its clone size (14) is therefore given by

$$P\left(Y_j=\left(a_j,d_j\right) \mid X_j=x_j,N_w\right)=\mathrm{b}\left(a_j;d_j,p\left(x_j,N_w\right)\right). \tag{17}$$

2.4.4 Likelihood of the clonal evolution of a single genetic time series

To compute the model evidence for $\mathcal{M}_{BD}$ we integrate the posterior distribution of the parameters to obtain

$$P\left(\mathcal{D}_m^i \mid \mathcal{M}_{BD}\right)=\int_s \sum_{N_w} P\left(s\right) P\left(N_w\right) P\left(Y_{1:n}^i \mid s,N_w\right) ds. \tag{18}$$

We choose the prior $P(s)$ to be uniform on $[0,0.5]$ and the prior $P(N_w)$ uniform on $[10\times\ 10^3,200\times\ 10^3]$.

2.4.5 Likelihood of the clonal evolution of several clones of the same mutation in different individuals

We now want to derive the probability of observing data across several participants $\mathcal{D}_m=\bigcup_{i\in\mathcal{I}_m}\mathcal{D}_m^i$ with the same mutation m. In this case, each trajectory has a set of independent model parameters (s_i,N_i) corresponding to the fitness of a genetic variant and the total number of wild-type stem cells in each individual. Here we allow the same mutation to have a different fitness in different individuals. In this case the evolution of clonal trajectories are independent for each participant and we obtain that

$$P\left(\mathcal{D}_m \mid \mathcal{M}_{BD}\right)=\prod_{i\in\mathcal{I}_m} P\left(\mathcal{D}_m^i \mid \mathcal{M}_{BD}\right). \tag{19}$$

2.4.6 Likelihood of the clonal evolution in the presence of other clones

If we know of other clones present in an individual, the allele proportion of a clone of size $X_m=k$ is given by

$$\hat{p}\left(k,N_w,X^i\right)=k/2\left(k+N_w+\sum_{j\neq m}X_j\right)=k/2\left(N_w+X^i\right), \tag{20}$$

where N_w is to the number of wild-type stem cells, $X^i=\sum_m X_m^i$ is the total size of fit clones in this individual.

The observation probability then becomes a joint probability in all clone sizes of the individual, but we may approximate this by assuming the size of the other clones present to be fixed at their average values, or equivalently taking the total clone size to be the sum of alternate observations, $X^i \approx p^{-1}(\sum_m a_m/d_m, N_w)$, so that the observation probability (17) is replaced by

$$P\left(Y_j = (a_j, d_j) \mid X_j = x_j, N_w\right) = \mathrm{b}\left(a_j; d_j, \hat{p}\left(x_j, N_w, X^i\right)\right). \tag{21}$$

The above makes the approximation that fluctuations in the different clones are not correlated.

2.4.7 Likelihood of the clonal evolution of multiple mutations on the same clone

Assume two mutations m, l are present on the same clone, and that the second mutation does not alter the fitness s of this clone. In other words, mutation l is a hitchhiker to m. To first approximation, a subclone's growth will only be affected by the average size of the other clones, but not their fluctuations. The approximation is similar to that made in (21), only that now the total number of stem cells $N_w + X^i$ will have to be recomputed to not count the size of the hitchhiking clone. In this case the observation probability for a mutation is approximately

$$P\left(Y_j = (a_j, d_j) \mid X_j = x_j, N_w\right) = \mathrm{b}\left(a_j; d_j, \hat{p}\left(x_j, N_w, \tilde{X}^i\right)\right). \tag{22}$$

$\tilde{X}^i$ is the total size of fit clones in this individual (incl. other mutations that may not be on the same clone as m or l), i.e., $\tilde{X}^i = X^i_m + X_{other} = X^i - X^i_l$. Note X^i_m (as used in preceding sections) quantifies the size of the clone with mutation m regardless of clonal substructure. In other words, we can use $p\left(k, N_w, X_i\right)$ as in section 2.4.6 if we ignore the counts of the smaller subclones, yielding $\tilde{X}^i$.

The likelihood of the two trajectories, $\mathcal{D}^i_{m,l} = \{Y^i_{1:n,m}, Y^i_{1:n,l}\}$, is approximately

$$P\left(\mathcal{D}^i_{m,l} \mid s, N_w, \mathcal{M}^{[m,l]}_{BD}\right) \approx P\left(Y^i_{1:n,m} \mid s, N_w\right) P\left(Y^i_{1:n,l} \mid s, N_w\right) \tag{23}$$

where the superscript [m,l] denotes that this model assumes both mutations on the same clone.

If these are the are the only two mutations in the individual the model evidence is

$$P\left(\mathcal{D}^i_{m,l} \mid \mathcal{M}^{[m,l]}_{BD}\right) \approx \int_s \sum_{N_w} P\left(s\right) P\left(N_w\right) P\left(Y^i_{1:n,m} \mid s, N_w\right) P\left(Y^i_{1:n,l} \mid s, N_w\right) ds. \tag{24}$$

2.4.8 Model comparison for clonal structure

To compare whether multiple mutations m, l in an individual are on separate clones ($\mathcal{M}_{BD}^{[m][l]}$) or the same clone ($\mathcal{M}_{BD}^{[m,l]}$), we compute the following model evidence for $\mathcal{M}_{BD}^{[m][l]}$

$$P\left(\mathcal{D}_{m,l}^{i} \mid \mathcal{M}_{BD}^{[m][l]}\right) = \sum_{N_w} P\left(N_w\right) \int_{s_m} P\left(s_m\right) P\left(Y_{1:n,m}^{i} \mid s_m, N_w, \tilde{X}^i\right) ds_m \cdot \int_{s_l} P\left(s_l\right) P\left(Y_{1:n,l}^{i} \mid s_l, N_w, \tilde{X}^i\right) ds_n, \quad (25)$$

and compare this to (24) using Bayes Factors. Since co-occurring mutations should be relatively rare, we bias against model $\mathcal{M}^{[m,l]}$ by increasing the prior of each model from 1 by 0.5 for each additional clone beyond the first one, i.e. in this case $P(\mathcal{M}^{[m,l]}) = 1$ and $P(\mathcal{M}^{[m][l]}) = 1.5$.

The above extends *mutatis mutandis* to the case of more than two mutations present on the same clone.

3 Inferring clonal fitness

The posterior distribution of fitness for a mutation m is given by

$$P\left(s \mid \mathcal{D}_m, \mathcal{M}_{BD}\right) = \sum_{N_w} P\left(\mathcal{D}_m \mid s, N_w, \mathcal{M}_{BD}\right) P(s) P(N_w) \quad (26)$$

where $P(\mathcal{D}_m \mid s, N_w, \mathcal{M}_{BD})$ is given by (19) (with (21)) or (23) in the case of multiple mutations on the same clone.

In the case of multiple clones, we marginalise over the fitness of all other clones to obtain the posterior distribution of fitness. For the case of two clones m and l, this results in

$$P\left(s_m \mid \mathcal{D}_m, \mathcal{M}_{BD}\right) = \sum_{N_w} P\left(\mathcal{D}_m \mid s_m, N_w, \mathcal{M}_{BD}\right) P(s_m) \int_{s_l} P\left(\mathcal{D}_l \mid s_l, N_w, \mathcal{M}_{BD}\right) P(s_l) P(N_w) ds_l \quad (27)$$

for the fitness distribution of clone m and analogously for fitness of clone l or the case of more than two clones.

Appendices

A A stochastic model of neutral clones

A.1 Evolution of HSPC counts in neutral clones

Assume that all HSPCs in an individual follow a critical birth-death (CBD) process ($s = 0$). When a mutation occurs in a HSPC it gives rise to a genetic clone c_m with

$$n_m(t) = \text{Number of HSPC cells in clone } c_t \text{ at time } t. \tag{28}$$

For now, we assume that mutations have no impact in the bias towards self-renewal, s, and that n_m in turn follows a CBD process. We refer to $\{c_m\}_{m \in \mathcal{C}}$ as the collection of all *neutral clones* ($s = 0$) present in the individual. If we denote by t_m the time of acquisition of the mutation, so that $n_m(t_m) = 1$, then the probability distribution of $n_m(t)$ is given by the limiting case $s \to 0$ of (4) , (Bailey, 1990). That is, for any $t > t_m$,

$$P(n_m(t) = k) = \begin{cases} \left(\frac{1}{1+\lambda(t-t_m)}\right)^2 \left(\frac{\lambda(t-t_m)}{1+\lambda(t-t_m)}\right)^{k-1}, & k > 0 \\ \frac{\lambda(t-t_m)}{1+\lambda(t-t_m)}, & k = 0, \end{cases} \tag{29}$$

Further, given $t_0 > t_m$, for any $t > t_0$ consider

$$p_k^{n_m}(t, a) := P(n_m(t + t_0) = k \mid n_m(t_0) = a). \tag{30}$$

The mean and variance of this probability distribution can be derived, again, as the limit $s \to 0$ of (8),

$$\mu_{n_m}(t) = n_m(t_0) \quad \text{and} \quad \sigma^2_{n_m}(t) = 2\lambda n_m(t_0)(t - t_0). \tag{31}$$

The distribution of neutral clones is heavily influenced by extinction events. Instead we turn out attention to the distribution of non-extinct neutral clones starting at time $t = 0$:

$$\tilde{p}_k^{n_m}(t) := P(n_m(t) = k \mid n_m(0) = 1, n_m(t) > 0). \tag{32}$$

The study of this process dates back to the work of Kendall (Kendall, 1948) and it can be shown (Tavaré, 2018) that for $k \geq 1$,

$$\tilde{p}_k^{n_m}(t) = nb(k - 1; 1, \beta(t)),$$

where nb is the probability mass function of the negative binomial distribution.

$$\beta(t) = \frac{\lambda t}{1 + \lambda t}.$$

Note that the distribution of surviving neutral clones starting at time $t = 0$ can be seen as a translated negative binomial and in what follows we will approximate the distribution of non-extinct neutral clones by NegBin $(1, beta(t))$ and use that

$$\mu_{n_i}(t) = \frac{\beta(t)}{(1-\beta(t))} = \lambda t \quad \text{and} \quad \sigma^2_{n_i}(t) = \frac{\beta(t)}{(1-\beta(t))^2} = \lambda t \, (1 + \lambda t) \,. \tag{33}$$

A.2 Distribution of VAF sizes in neutral clones

Next, we want to understand how the stochastic evolution of HSPC counts over time translates to the evolution of the variant allele frequency (VAF or blood share) of genetic clones.

A.2.1 Linear scaling of random variables

First, let us remind the scaling properties of the mean and variance of a random variable under affine transformations. Let X be a random variable with mean μ_X and variance σ^2_X and define Y as

$$Y = \frac{X + b}{c}, \tag{34}$$

for scalars b and c. Then mean and variance scale as follows,

$$\mu_Y = \frac{\mu_X + b}{c} \quad \text{and} \quad \sigma^2_Y = \frac{\sigma^2_X}{c^2}. \tag{35}$$

A.2.2 Distribution of VAF evolution

Since we assume that all HSPCs in the individual follow a CBD process, the total population of HSPCs remains stable, on average, at N. Further, since asymmetric divisions are far more common than symmetric divisions, we can assume that the number of differentiated blood cells produced by a clone of HSPCs is directly proportional to the clone's size. We therefore model the VAF of a clone c_m at time $t > t_m$ as

$$v_m(t) = \frac{n_m(t)}{2N}, \tag{36}$$

where the factor 2 in the denominator is the result of diploidy in HSPCs. It follows from the distribution of non-extinct clone sizes (33) and scaling properties

(35) that the mean and variance of the VAF distribution of non-extinct clone clones starting at time $t = 0$ is

$$\mu_{v_m}(t) = \frac{\lambda t}{2N} \quad \text{and} \quad \sigma^2_{v_m}(t) = \frac{\lambda t\,(1+\lambda t)}{4N^2}. \tag{37}$$

A.3 Maximum size of neutral clones

Given an individual aged t, we can then estimate the maximum size genetic clones without any fitness advantage can reach. We estimate this by taking 2 standard deviations from the mean of the evolution of non-extinct clones following a CBD process starting at age 0:

$$\max_m v_m(t) = \mu_v(t) + 2\sigma_v(t),$$

assuming that v initiated at time $t = 0$ and $v(0) = 1/2N$.

It then follows from (37) that

$$\max_m v_m(t) = \frac{\lambda t + 2\sqrt{\lambda t(1+\lambda t)}}{2N},$$

and that for long times, $t \gg 1$,

$$\max_m v_m(t) = \frac{\lambda}{N} O(t).$$

Let us recall that current estimates place $\lambda/N \sim 10^{-5}$ per year. We can therefore place an estimate for the maximum size of neutral clones in a participant aged 100 years at

$$\max_m v_m(t) \approx 0.001.$$

B Deterministic model of VAF evolution

Instead of using a stochastic process as we have done in our inference pipeline, one can alternatively resort to the deterministic limit of a birth-death process to model the longitudinal evolution of clonal dynamics, as detailed in (3).

Under this model, a population of cells, with mutation m, grows exponentially as

$$X_m(t) = e^{s_m(t-t_m)}, \tag{38}$$

for any $t > t_m$, where t_m is the time of mutation and s_m its associated fitness. Note that this model is a continuous model that no longer accurately reflects the discrete nature of cell divisions.

Analogously to (20), we assume that individuals have N_w wild-type stem cells following a critical birth-death process, i.e., with zero fitness. N_w therefore remains constant in time. The total number of stem cells, $N(t)$, in the individual then grows as

$$N(t) = N_w + X_m(t).$$

Consequently, the VAF, v_m, associated to clone c_m displays a logistic growth with time,

$$v_m(t) = \frac{X_m(t)}{2N(t)}. \tag{39}$$

Note that this setup is easy to generalise to account for more than one mutation coexisting in an individual. If an individual has a collection of $\{m\}_{m \in \mathcal{M}}$ fitness inducing mutations, the number of stem cells in this individual grows as

$$N(t) = N_w + \sum_m X_m(t), \tag{40}$$

and $v_m(t)$ is computed analogously to (39).

This model can easily be extended to account for coexisting mutations on the same clone. Consider the case were two mutations m and l, harnessed at times $t_m < t_l$, are present in the same clone and assume that the secondary mutation does not increase the fitness of the clone, so that they share a common fitness s. It is then clear from (40) that the deterministic evolution of $N(t)$ is dictated by the evolution of the earlier mutation,

$$N(t) = N_w + X_m(t). \tag{41}$$

The generalisation of $N(t)$ to the case of several groups of mutations coexisting in clones in an individual is easily derived *mutatis mutandis*.

B.1 Maximum likelihood fitting

In order to fit the deterministic model to longitudinal data, one should also take into account the sequencing error in the data. To this end, we again use the binomial model of sampling noise, as in (17).

Consider the time-series data, $Y_{1:n}$, associated to a singly occurring mutation in a participant, analogous to the notation set in (10). Further recall that each data point, $Y_j = (a_j, d_j, t_j)$, is characterised by the read depth and alternate

observations of a genetic mutation, a_j and d_j respectively, as well as the time of observation t_j.

Given an initial time of mutation, t_0, fitness, s, and total number of wild-type cells, N_w, we model the probability of observing data points by

$$P\left(Y_j \mid t_0, s, N_w\right) = b\left(a_j; d_j, v(t_j; t_0, s, N_w)\right), \tag{42}$$

where b corresponds to the probability mass function of the binomial distribution and $v(t_j)$ to the deterministic VAF associated to the mutation,

$$v(t_j; t_0, s, N_w) = \frac{e^{s(t_j - t_0)}}{2(N_w + e^{s(t_j - t_0)})}, \tag{43}$$

as in (39).

One can then simply use maximum likelihood estimation to find the optimal parameters associated to the data associated to a mutation. Again, this framework extends to the case of more than one mutation and coexistence of mutations in clones using the appropriate formulation of $v(t)$ as detailed above.

B.2 Visualising stochastic fits using the deterministic model

One can also use the deterministic framework to visually asses the "goodness of fit" of the *maximum a posteriori* (MAP) estimates obtained using the Hidden Markov model detailed in Section 3. To this end, we fix parameters s and N_w to the MAP estimates and fit only the time of mutation, t_0.

Recall that the Hidden Markov model allows us to obtain the posterior distribution of fitness, s, and wild-type stem cells, N_w, for a single mutation occurring in an individual. We can then use the MAP inferred values, $\overline{s}$ and $\overline{N_w}$, as fixed parameters of the deterministic model. The probability of observing a data point Y_j then becomes function of a single parameter, t_0,

$$P\left(Y_j \mid t_0, s, N_w\right) = b\left(a_j; d_j, v(t_j; t_0, \overline{s}, \overline{N_w})\right). \tag{44}$$

Using maximum likelihood to fit the time of origin of the mutation, $\overline{t_0}$, will then produce a deterministic evolution of $v(t, \overline{t_0}, \overline{s}, \overline{N_w})$, that can be used a visual assessment of the MAP estimates of fitness and wilt-type stem cells.

References

N. T. Bailey. *The elements of stochastic processes with applications to the natural sciences*, volume 25. John Wiley & Sons, 1990.

Z. Ghahramani. An introduction to hidden markov models and bayesian networks. In *Hidden Markov models: applications in computer vision*, pages 9–41. World Scientific, 2001.

D. G. Kendall. On the generalized "birth-and-death" process. *The Annals of Mathematical Statistics*, 19(1):1–15, mar 1948. doi: 10.1214/aoms/1177730285.

I. Martincorena, A. Roshan, M. Gerstung, P. Ellis, P. V. Loo, S. McLaren, D. C. Wedge, A. Fullam, L. B. Alexandrov, J. M. Tubio, L. Stebbings, A. Menzies, S. Widaa, M. R. Stratton, P. H. Jones, and P. J. Campbell. High burden and pervasive positive selection of somatic mutations in normal human skin. *Science*, 348(6237):880–886, may 2015. doi: 10.1126/science.aaa6806.

S. Tavaré. The linear birthâdeath process: an inferential retrospective. *Advances in Applied Probability*, 50(A):253–269, dec 2018. doi: 10.1017/apr.2018.84.

J. E. Till, E. A. McCulloch, and L. Siminovitch. A stochastic model of stem cell proliferation, based on the growth of spleen colony-forming cells. *Proceedings of the National Academy of Sciences of the United States of America*, 51(1): 29, 1964.

C. J. Watson, A. Papula, G. Y. Poon, W. H. Wong, A. L. Young, T. E. Druley, D. S. Fisher, and J. R. Blundell. The evolutionary dynamics and fitness landscape of clonal hematopoiesis. *Science*, 367(6485):1449–1454, 2020.
