# Supplementary material for: Longitudinal dynamics of clonal hematopoiesis identifies gene-specific fitness effects
Source: Nat Med. 2022 Jul 4;28(7):1439–46. doi: 10.1038/s41591-022-01883-3 (PMC9307482; doi:10.1038/s41591-022-01883-3)
Supplement: Supplementary file 2 — Reporting Summary [file 41591_2022_1883_MOESM2_ESM.pdf]

nature research

Corresponding author(s): Linus Schumacher, Kristina Kirschner, Tamir Chandra

Last updated by author(s): Jan 5, 2022

Reporting Summary

Nature Research wishes to improve the reproducibility of the work that we publish. This form provides structure for consistency and transparency in reporting. For further information on Nature Research policies, see our Editorial Policies and the Editorial Policy Checklist.

Statistics

For all statistical analyses, confirm that the following items are present in the figure legend, table legend, main text, or Methods section.

n/a	Confirmed	
☐	☒	The exact sample size (*n*) for each experimental group/condition, given as a discrete number and unit of measurement
☒	☐	A statement on whether measurements were taken from distinct samples or whether the same sample was measured repeatedly
☐	☒	The statistical test(s) used AND whether they are one- or two-sided<br>*Only common tests should be described solely by name; describe more complex techniques in the Methods section.*
☐	☒	A description of all covariates tested
☐	☒	A description of any assumptions or corrections, such as tests of normality and adjustment for multiple comparisons
☐	☒	A full description of the statistical parameters including central tendency (e.g. means) or other basic estimates (e.g. regression coefficient) AND variation (e.g. standard deviation) or associated estimates of uncertainty (e.g. confidence intervals)
☐	☒	For null hypothesis testing, the test statistic (e.g. *F*, *t*, *r*) with confidence intervals, effect sizes, degrees of freedom and *P* value noted<br>*Give P values as exact values whenever suitable.*
☐	☒	For Bayesian analysis, information on the choice of priors and Markov chain Monte Carlo settings
☒	☐	For hierarchical and complex designs, identification of the appropriate level for tests and full reporting of outcomes
☐	☒	Estimates of effect sizes (e.g. Cohen's *d*, Pearson's *r*), indicating how they were calculated

Our web collection on statistics for biologists contains articles on many of the points above.

Software and code

Policy information about availability of computer code

Data collection

No software was used for data collection

Data analysis

All the code developed for the data analysis of this article is publicly available and documented in the Github repository: https://github.com/neilrobertson/LBC_ARCHER

Workflow overview
A workflow chart describing the full pipeline and implementation guidance are included in the github repository (see Code Availability). Our pipeline can be applied to other datasets with a few adjustments. Our LiFT algorithm has been tailored to the LBC dataset by extracting parameters from the distribution of synonymous mutation reads which inform the priors used for our Bayesian inference method (see SI methods Section 2.3.3 and Extended Data Fig. A, B and C). Guidance on how to adapt our LiFT algorithm to other datasets is included in the code repository. All other parts of the pipeline, including the extraction of variants using ArcherDx software and the inference of clonal structures and fitness, are directly applicable to other datasets.

Data analysis is split in 3 parts:

1) Variant calling, data aggregation and quality control
* Variant calling and raw data processing was completed using the ArcherDX/Invitea analysis pipeline. This was received as a virtual machine that was hosted within the University of Edinburgh (Virtualisation Team). https://analysis.archerdx.com/static/Archer_Analysis_Manual_4_1_0.pdf
* Data aggregation was performed using python and R scripts contained in a sub-folder within the Git repository.
Data curation was undertaken in Python v.3.7 and R base with use of the “tidyverse” suite of packages and plotted with ggplot2.

2) Longitudinal analysis of variants.
Longitudinal analysis of variants is implemented in Python v.3.7 with dependencies on Numpy v.1.21.5, Scipy v.1.7.3 and Pandas 1.3.4.
Survival analysis was implemented using Python v.3.7 with dependencies on Lifelines 0.26.4.

3) Survival analysis.
Survival analysis is implemented in Python programming language using Lifelines package.

For manuscripts utilizing custom algorithms or software that are central to the research but not yet described in published literature, software must be made available to editors and reviewers. We strongly encourage code deposition in a community repository (e.g. GitHub). See the Nature Research guidelines for submitting code & software for further information.

Data

Policy information about availability of data

All manuscripts must include a data availability statement. This statement should provide the following information, where applicable:

- Accession codes, unique identifiers, or web links for publicly available datasets
- A list of figures that have associated raw data
- A description of any restrictions on data availability

We have deposited all data pertinent to this analysis including the de-identified raw fastq read data and processed variant calls for our longitudinal cohort onto the NCBI Gene Expression Omnibus (Geo) with accession ID: GSE178936 (https://www.ncbi.nlm.nih.gov/geo/query/acc.cgi?acc=GSE178936). LBC phenotypic data are available at dbGAP under the accession number phs000821.v1.p1 (https://www.ncbi.nlm.nih.gov/projects/gap/cgi-bin/study.cgi?study_id=phs000821.v1.p1).

All other Lothian Birth Cohort Data are deposited in dbGAP or provided via the LBC DAC (https://www.ed.ac.uk/lothian-birth-cohorts/data-access-collaboration). Information concerning the cohort is contained here: including its history, data summary tables for both LBC1921 and LBC1936 with data access request forms and contact information to obtain all data points.

Field-specific reporting

Please select the one below that is the best fit for your research. If you are not sure, read the appropriate sections before making your selection.

☒ Life sciences ☐ Behavioural & social sciences ☐ Ecological, evolutionary & environmental sciences

For a reference copy of the document with all sections, see nature.com/documents/nr-reporting-summary-flat.pdf

Life sciences study design

All studies must disclose on these points even when the disclosure is negative.

Sample size	Sample size was not estimated but samples were selected on the basis of harbouring CHIP variants in previous whole genome sequencing (WGS) at wave one. Our methodology is designed to determine fitness estimates in single samples, ergo, sample size was deemed sufficient.
Data exclusions	The study was initially focused on healthy cognitive ageing; at the inception of the study (at approximately age 70), participants were recruited if they reported no dementia or other neurodegenerative diagnoses. In addition, to take part in the first wave of the study, participants had to have been born in 1936 in Scotland, and be living in the Edinburgh and Lothians area of Scotland when recruited.<br><br>Some data-points
Replication	not applicable
Randomization	not applicable
Blinding	not applicable

Reporting for specific materials, systems and methods

We require information from authors about some types of materials, experimental systems and methods used in many studies. Here, indicate whether each material, system or method listed is relevant to your study. If you are not sure if a list item applies to your research, read the appropriate section before selecting a response.

Materials & experimental systems

n/a	Involved in the study
☒	☐ Antibodies
☒	☐ Eukaryotic cell lines
☒	☐ Palaeontology and archaeology
☒	☐ Animals and other organisms
☐	☒ Human research participants
☒	☐ Clinical data
☒	☐ Dual use research of concern

Methods

n/a	Involved in the study
☒	☐ ChIP-seq
☒	☐ Flow cytometry
☒	☐ MRI-based neuroimaging

Human research participants

Policy information about studies involving human research participants

Population characteristics

The Lothian Birth Cohort 1921 (LBC1921) contains a total of 550 healthy participants at Wave 1 of their testing (done between 1999 and 2001) with a gender ratio of 234/316 (m/f) and a mean age at Wave 1 of 79.1 (SD=0.6). The Lothian Birth Cohort 1936 (LBC1936), contains a total of 1091 healthy participants at Wave1 of their testing (done between 2004 and 2007) with a gender ratio of 548/543 (m/f) and a mean age at Wave 1 of 69.5 (SD=0.8) (Taylor et al., 2018)). Both cohorts are Scottish cohorts. Participant characteristics of the whole cohort are described in the articles cited in the box directly below. Participants characteristics of the specific subsample used in the present study are described in the Methods section of the manuscript. There is known range restriction among members of LBC1921 and LBC1936. They were healthier and better educated than members of the general population of the same age, e.g. Johnson et al 2011 Health Psychology 30:1-11.

Recruitment

Recruitment for LBC1921 was similar to LBC1936.
The Lothian Birth Cohort 1921 participants were identified for invitation to participate via newspaper advertisements and also via linkage with the NHS to identify addresses of those individuals born in 1921 living in the region (most schoolchildren in 1932 had taken the Scottish Mental Survey 1932 at school, and the study was designed as a follow-up of those older adults, aged ~79 at recruitment).
The Lothian Birth Cohort 1936 participants were identified for invitation to participate via newspaper advertisements and also via linkage with the NHS to identify addresses of those individuals born in 1936 living in the region (most schoolchildren in 1936 had taken the Scottish Mental Survey 1947 at school, and the study was designed as a follow-up of those older adults, aged ~70 at recruitment. The study protocol papers that describe this recruitment process - along with the ethical approvals - are described in detail in the following open access protocol papers:
https://www.ncbi.nlm.nih.gov/pmc/articles/PMC2222601/
https://pubmed.ncbi.nlm.nih.gov/22253310/
https://www.ncbi.nlm.nih.gov/pmc/articles/PMC6124629/

Ethics oversight

Ethics permission for the Lothian Birth Cohort 1936 was obtained from the Multi-Centre Research Ethics Committee for Scotland (Wave 1: MREC/01/0/56), the Lothian Research Ethics Committee (Wave 1: LREC/2003/2/29), and the Scotland A Research Ethics Committee (Waves 2, 3, 4 & 5: 07/MRE00/58). Ethics permission for the Lothian Birth Cohort 1921 (LBC1921) was obtained from the Lothian Research Ethics Committee (Wave 1: LREC/1998/4/183; Wave 2: LREC/2003/7/23; Wave 3: 1702/98/4/183) and the Scotland A Research Ethics Committee (Waves 4 and 5: 10/MRE00/87).

Note that full information on the approval of the study protocol must also be provided in the manuscript.
